# Bow-tie signaling in c-di-GMP: Machine learning in a simple biochemical network

**DOI:** 10.1371/journal.pcbi.1005677

**Published:** 2017-08-02

**Authors:** Jinyuan Yan, Maxime Deforet, Kerry E. Boyle, Rayees Rahman, Raymond Liang, Chinweike Okegbe, Lars E. P. Dietrich, Weigang Qiu, Joao B. Xavier

**Affiliations:** 1 Program for Computational and Systems Biology, Memorial Sloan-Kettering Cancer Center, New York, NY, United States of America; 2 Department of Biological Sciences, Hunter College & Graduate Center, CUNY, New York, NY, United States of America; 3 Department of Biological Sciences, Columbia University, New York, NY, United States of America; Rice University, UNITED STATES

## Abstract

Bacteria of many species rely on a simple molecule, the intracellular secondary messenger c-di-GMP (Bis-(3'-5')-cyclic dimeric guanosine monophosphate), to make a vital choice: whether to stay in one place and form a biofilm, or to leave it in search of better conditions. The c-di-GMP network has a bow-tie shaped architecture that integrates many signals from the outside world—the input stimuli—into intracellular c-di-GMP levels that then regulate genes for biofilm formation or for swarming motility—the output phenotypes. How does the ‘uninformed’ process of evolution produce a network with the right input/output association and enable bacteria to make the right choice? Inspired by new data from 28 clinical isolates of *Pseudomonas aeruginosa* and strains evolved in laboratory experiments we propose a mathematical model where the c-di-GMP network is analogous to a machine learning classifier. The analogy immediately suggests a mechanism for learning through evolution: adaptation though incremental changes in c-di-GMP network proteins acquires knowledge from past experiences and enables bacteria to use it to direct future behaviors. Our model clarifies the elusive function of the ubiquitous c-di-GMP network, a key regulator of bacterial social traits associated with virulence. More broadly, the link between evolution and machine learning can help explain how natural selection across fluctuating environments produces networks that enable living organisms to make sophisticated decisions.

## Author summary

How does evolution shape living organisms that seem so well adapted that they could be intelligently designed? Here, we address this question by analyzing a simple biochemical network that directs social behavior in bacteria; we find that it works analogously to a machine learning algorithm that learns from data. Inspired by new experiments, we derive a model which shows that natural selection—by favoring biochemical networks that maximize fitness across a series of fluctuating environments—can be mathematically equivalent to training a machine learning model to solve a classification problem. Beyond bacteria, the formal link between evolution and learning opens new avenues for biology: machine learning is a fast-moving field and its many theoretical breakthroughs can answer long-standing questions in evolution.

## Introduction

**Fig 1:**
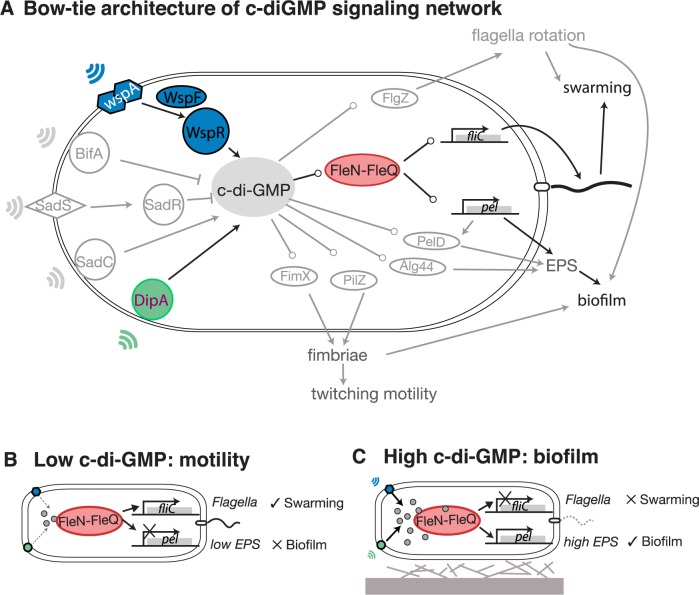
Bacteria integrate stimuli from the environment and decide whether to make biofilms or to move using the c-di-GMP network. A: Bow-tie architecture of c-di-GMP signaling network: c-di-GMP is synthesized by diguanylate cyclase (DGC) proteins with GGDEF domains such as WspR, DipA, and SadC, and degraded by phosphodiesterases (PDE) proteins with EAL or HD-GYP domains such as BifA, and SadR. The DGCs and PDEs could sense stimuli—such as chemoattractants which could be a signal for motility, or mechanical contact with surfaces which could be a signal for biofilm formation—and change intracellular c-di-GMP levels in response; c-di-GMP effectors—such as c-di-GMP binding proteins and riboswitch RNAs—then sense c-di-GMP levels and control phenotype outputs such as biofilm formation, motility, virulence and cell division. B: At low levels of c-di-GMP the bacteria express flagella genes and go into motile mode. C: At high levels of c-di-GMP the bacteria repress flagella genes, express biofilm genes and go into biofilm mode.

Cells use networks of biochemical reactions to collect cues from the world around them, process that information internally and respond appropriately [1]. Understanding how evolution by natural selection has turned biochemical reactions into information-processing circuits remains a major challenge [2]. The intracellular secondary messenger c-di-GMP (Bis-(3'-5')-cyclic dimeric guanosine monophosphate), ubiquitous in bacteria, is a network hub lying at the core of signaling pathways with dozens of inputs and outputs. This type of network is called a bow-tie because of its shape (**Fig 1A**) [3]. The key feature of a bow-tie is its ability to compress multiple inputs and command multiple outputs [4]. We find bow-ties in cells that do sophisticated information processing. For instance, macrophages and dendritic cells that integrate toll-like receptor signals to decide on immune responses [5], and a neuron must integrate multiple stimuli into sequences of action potentials which it then delivers to several other neurons [6]. What is the function of the c-di-GMP bow-tie architecture in the bacterial cell?

We investigated this question in *Pseudomonas aeruginosa*. Like other bacteria [7], *P*. *aeruginosa* uses c-di-GMP to decide whether to stay in a place and form a biofilm, or to swarm away in search of better conditions. Biofilm formation is a social behavior in which bacteria attach to surfaces, secrete polymeric substances and form protective communities that make infections hard to treat with antibiotics [8,9]. Swarming is also a social behavior, but swarms are motile and biofilms are sessile; the two behaviors are mutually exclusive and require expressing different sets of genes [10]. A better understanding of how the *P*. *aeruginosa* cell commands biofilm and swarming behaviors could lead to anti-biofilm therapies against this major pathogen [11].

*P*. *aeruginosa* has dozens of proteins that make and break c-di-GMP. Diguanylate cyclase (DGC) proteins with GGDEF domains synthesize c-di-GMP, and phosphodiesterase (PDE) proteins with EAL or HD-GYP domains degrade c-di-GMP. DGCs and PDEs can respond to diverse stimuli such as contact with a surface or the presence of a chemical attractant. They modulate intracellular levels of c-di-GMP that then regulate expression of downstream genes [7]. According to a well-established model, when c-di-GMP levels are low the enhancer-binding protein FleQ activates flagella genes needed for swarming motility and represses extracellular matrix genes needed for biofilm formation [12,13] (**Fig 1B**). When c-di-GMP levels are high FleQ forms a complex with another protein, FleN, and the FleN-FleQ complex converts its function to repress flagella genes and de-repress biofilm matrix genes [14] (**Fig 1C**). The FleN-FleQ is therefore a c-di-GMP-responsive switch that creates an opposed co-regulation of biofilm and motility genes.

Co-regulation is efficient because *P*. *aeruginosa* cannot move and stay encased in a matrix at the same time [15], but it comes with a risk: Experimental evolution in swarming conditions selects for FleN mutants with many flagella called hyperswarmers, which are locked in a perpetual motile mode and cannot make proper biofilms [16]. This tradeoff between biofilms and swarming—a dichotomy due to their co-regulation by c-di-GMP—could be exploited in therapies against *P*. *aeruginosa* infections. However, two key obstacles remain: First, we lack systems-level understanding of the c-di-GMP network. We know reasonably well how some network components work—for example, physical contact with a solid surface stimulates the Wsp transmembrane complex to synthesize c-di-GMP [12,17]—but we know little about how they work together as a network [18]. Second, we know little about the network’s diversity across the *P*. *aeruginosa* species. The link between c-di-GMP, biofilm and swarming was repeatedly validated in isogenic mutants [19] but seems to be absent when compared across different strains [16,20]. Is the tradeoff really absent outside the laboratory, or is it buried by many genetic differences accumulated between strains since their common ancestor? Understanding how selective pressures shape the c-di-GMP network is crucial to new therapies, especially to prevent the emergence of resistance.

Here, we combined genomics, experimental evolution and mathematical modeling to elucidate the function of the c-di-GMP network. We investigated *P*. *aeruginosa* isolates from acutely infected cancer patients; this population is distinct from isolates from chronic infections, such as those formed in cystic fibrosis lungs where microbial strains already experienced long-term evolution within the host [21–25]. Against our expectations, we saw no correlation between c-di-GMP, biofilm and swarming levels. To explain these observations, we developed a mathematical model from biochemical reaction principles; we derived a mechanism of how selection across fluctuating environments can tune the c-di-GMP network analogous to machine learning. The model explains why fluctuating environments, such as natural systems and short-term infections, could select for generalist strains but stable environments, such as laboratory evolution or long-term infections, could select for specialists locked in a phenotypic mode. We then applied our knowledge to directed-evolution experiments that revealed new mutations causing loss of biofilm specialism.

## Results

### *P*. *aeruginosa* clinical isolates: High diversity in c-di-GMP, swarming and biofilm formation with no apparent correlation

**Fig 2:**
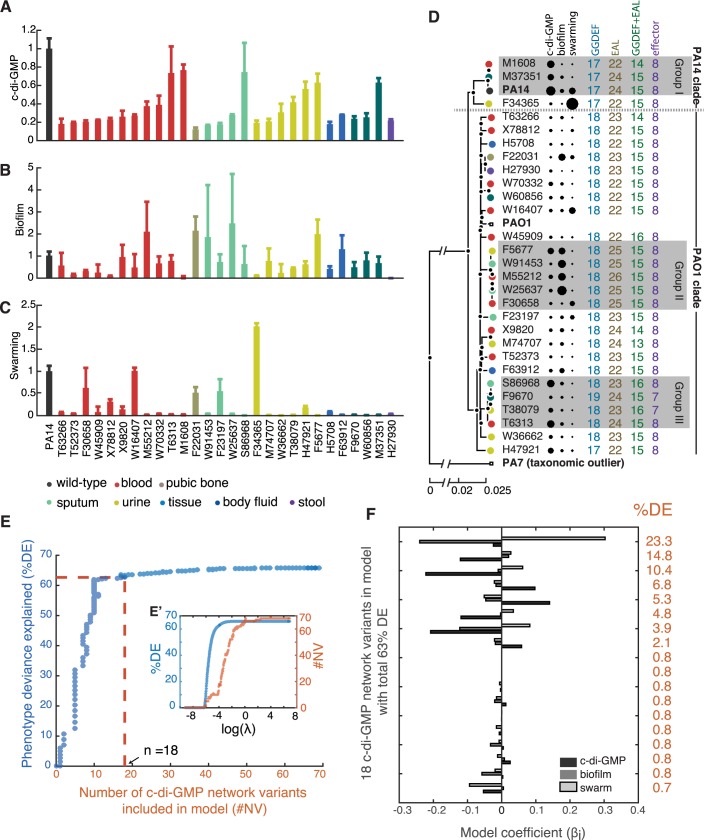
Phenotypic diversity in 28 *P*. *aeruginosa* isolates from acutely infected cancer patients at MSKCC explained by many small-effect alleles in c-di-GMP network. A: Bulk c-di-GMP levels collected from bacterial colonies, including for the laboratory strain PA14. B: Biofilm levels measured in microtiter plates using the crystal-violet assay. C: Motility measured as swarm area after 16 h of incubation. D: Phylogenetic tree reconstructed from 88,347 genetic variants identified in core genes, including PA14 and two other laboratory strains PAO1 and PA7. Numbers shown represent the number of open-reading frames (ORFs) identified with c-di-GMP related motifs: GGDEF domain for synthesizing c-di-GMP, EAL for degrading c-di-GMP, and effector for sensing c-di-GMP. Some ORFs encode both GGDEF and EAL domains. E: Explaining diversity in c-di-GMP, biofilm and swarming required many alleles of small-effect in c-di-GMP genes identified within the 28 genomes. Model selection using LASSO revealed that a model that explains 85% of the phenotypic deviance requires including at least 21 genetic variants in c-di-GMP related genes. E’ shows a detail of LASSO model selection, which increases the tuning parameter λ and selects variants to include in the model. F: Each of the 21 genetic variants by itself explains 27% or less of the phenotypic variance, even in the best model selected by LASSO. The analysis supports that the phenotypic diversity observed among clinical isolates is due to small-effect alleles.

We selected a cohort of 28 clinical isolates of *P*. *aeruginosa* to investigate associations between c-di-GMP and two social phenotypes—biofilm formation and swarming—that it regulates. The 28 strains originated from a diversity of sample types (blood, urine, etc.) obtained from acutely infected patients at Memorial Sloan Kettering Cancer Center (MSKCC), and belonged to a larger set of *P*. *aeruginosa* strains that—we had described before [16]—vary in their capacity for biofilm formation and swarming. To understand how the diverse levels of biofilm formation and swarming relate to c-di-GMP, we measured each strain’s bulk c-di-GMP levels from extracts obtained from dense colonies grown on Petri dishes [26]. The c-di-GMP levels varied significantly between the isolates and from those measured for the laboratory strain PA14 (**Fig 2A**, p<0.05). We found no association between the c-di-GMP level and the sample type (blood, urine, etc., p>0.05), and also no correlation between c-di-GMP and biofilm formation (quantified by the microtiter crystal violet assay [27]) or swarming motility (quantified by the colony area at 16 h [16], **Figs 2B, 2C, S1A and S1B**). The two social phenotypes also did not correlate with each other (**Fig A** in **S2 Fig**. p>0.05).

The apparent lack of correlations seemed to challenge the well-established notion that c-di-GMP imposes a tradeoff between biofilm and swarming [28,29]. Another explanation, however, was that the 28 strains, despite coming from the same hospital, might be phylogenetically diverse. *P*. *aeruginosa* may live asymptomatically with its human host until immune-compromising cancer therapy facilitates opportunistic infection [30]; if the 28 strains spanned a large phylogenetic distance, the tradeoff could be hidden by many genetic differences accumulated during their separate evolutionary histories. To clarify this issue, we sequenced the whole-genomes of the 28 MSKCC isolates and reconstructed their phylogeny (**Fig 2D**). We included, for reference, the publicly available genome of PA14 and those of two other well-characterized strains, PAO1 and PA7 [31]. The phylogenetic tree confirmed features seen before—PA14 and PAO1 resided in two major clades [32] and PA7 was an outlier [33]—and revealed that the 28 isolates were indeed phylogenetically diverse from each other. Interestingly, the ability to infect a specific body site was not restricted by phylogeny: isolates from different sample types were found in both the PA14 and the PAO1 clades (circle colors, **Fig 2D**).

We then analyzed c-di-GMP levels, biofilm levels and swarming motility in the light of the reconstructed phylogeny. The sequenced genomes revealed that the strains varied little in the number of genes predicted to be in the c-di-GMP pathway (numbers listed next to each isolate, **Fig 2D**). A statistical analysis of phylogenetic signal, the Moran I test [34], indicated that the c-di-GMP level had a strong phylogenetic signal (p<0.05; **Fig A in S3 Fig**) but biofilm and swarming had not (p>0.05; **Fig B,C in S3 Fig**). We then tried correlating biofilm and swarming using the method of phylogenetic generalized least squares regression (PLSR) [35]—a method that correlates two phenotypes after correcting for phylogeny (**see S1 Text**). PLSR showed a significant anti-correlation (**Fig B in S2 Fig**) which would support a tradeoff between biofilm and swarming. But the anti-correlation depended on a subset of three strains—M37351, M55212 and F30658—that were closely related and had strong phenotypic differences among them. The correlation vanished if we excluded those three strains from the analysis (**Fig C in S2 Fig**), which indicates that the tradeoff between biofilm formation and swarming is hard to detect across large phylogenic distances. We investigated the correlation between biofilm and swarming in three groups of closely related clinical isolates after PLSR (**Fig 2D**, gray shaded). The genomes in those three subgroups differ in 480, 593 and 1654 SNPs, respectively. The phylogenetically-corrected values of biofilm and swarming showed strong correlations in group I and II (**Fig AB in S4 Fig**) but not in group III (**Fig C in S4 Fig**). Other than phylogenetic distance, the correlations also depended on the phenotypic diversity observed in each groups. For example, F30658 in group II was a strong swarmer and weak biofilm-former—the opposite from the other strains of this subgroup. But all of the four strains in group III showed very similar phenotypes to each other. PLSR helped reveal the hidden correlation between biofilm and swarming, and supported that there is a tradeoff between the two co-regulated phenotypes but only among strains that are closely related and have different phenotypes.

We then investigated whether the pattern of c-di-GMP levels, biofilm and swarming observed across the entire phylogenetic tree could be explained by a few genetic variants of large effect in c-di-GMP network genes, or if explaining the pattern required many genetic variants of small effect. We used LASSO technique [36], an algorithm that searches for a small number of features to explain a set of observables (**see S1 Text**). We selected the smallest subset of genetic variants (the features) as we increased a penalization, λ, for including many features (**see S1 Text**). According to this analysis, explaining 63% of the phenotype deviance required a model with at least 18 variants in c-di-GMP network genes (**Fig 2E**). All variants were predicted to have low effect, since even the strongest variant would only explain 23.3% of the phenotypic deviance (**Fig 2F**). In summary, LASSO showed that c-di-GMP, biofilm and swarming—in addition to being uncorrelated when investigated across the entire tree—have a complex diversity that may not be explained by a small set of genetic alterations of large effect, but was more likely to result from a combination of genetic alterations of small effect.

### Biochemical reaction model of c-di-GMP network

**Fig 3:**
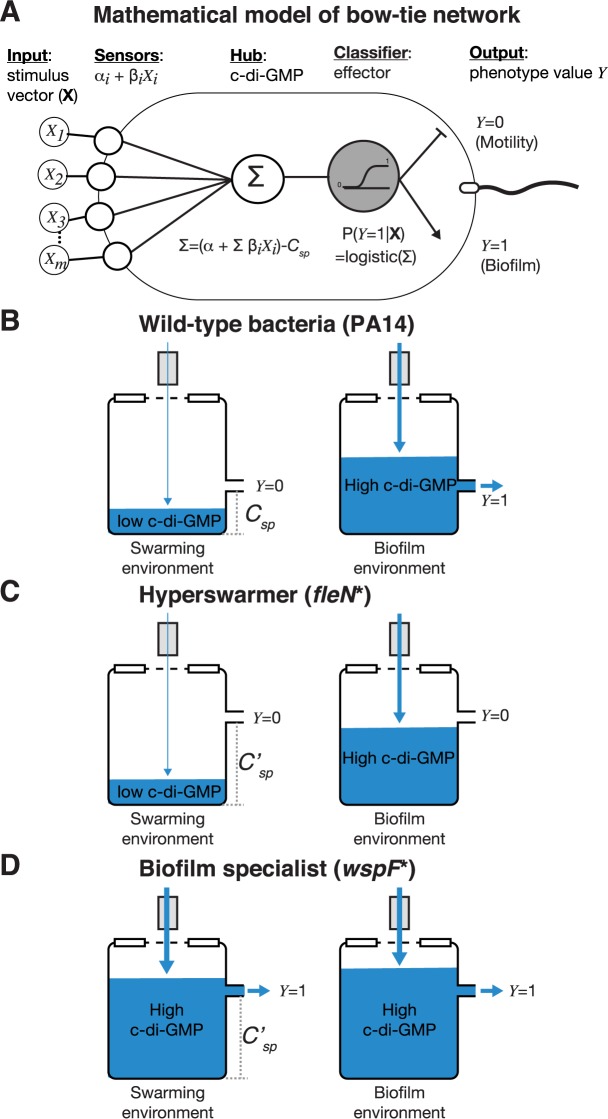
Bow-tie model of biochemical reactions in the c-di-GMP network explains mutants of PA14 evolved in the laboratory. A: Diagram of the bow-tie model showing the *α* and *β* coefficients for sensor and effector modules. B-D: A water tank diagram explains how the relative values of c-di-GMP and the effector setpoint lead to the specialist phenotypes for mutants (C,D) evolved from a generalist laboratory strain (PA14) in laboratory experiments (B).

The lack of correlations between c-di-GMP and the two social phenotypes that it commands—biofilm and swarming—raised an important question: how can the c-di-GMP network co-regulate those phenotypes and, at the same time, allow them to be uncorrelated across the phylogenetic tree? We sought to address this question with a simple theoretical model. The model considers that a bacterial cell has *m* biochemical sensors that can modulate intracellular c-di-GMP levels in response to environmental stimuli (**Fig 3A**). Each sensor is either a DGC (which synthetizes c-di-GMP) or a PDE (which degrades c-di-GMP), and we modeled their biochemical kinetics with commonly used methods (e.g. [37]):

Each DGC-based sensor synthetizes c-di-GMP (*C*) from its substrate—which the model assumes is non-limiting (represented by ∅)—with a basal synthesis rate, *R*_*i*,*basal*_. The rate increases to *R*_*i*,*basal*_ + *R*_*i*_ when the sensor binds to a cognate stimulus *X*_*i*_, which we modeled as a binary variable (*X*_*i*_ = 0 means the stimulus is absent, *X*_*i*_ = 1 means the stimulus is present). The reaction for DGC-based synthesis of c-di-GMP was therefore

$$\emptyset \overset{r_{i}}{\to }C\;where\;r_{i}=R_{i,basal}+R_{i}X_{i}$$

Similarly, a PDE-based sensor degrades c-di-GMP into a product—which we assumed does not affect the relevant kinetics (again represented by ∅)—at a basal consumption rate *R*_*j*,*basal*_*C*. The degradation rate goes to *R*_*j*,*basal*_*C* + *R*_*j*_ when the sensor binds to a cognate stimulus *X*_*j*_, which we also modeled as a binary variable. The reaction for PDE-based degradation of c-di-GMP was therefore

$$C\overset{r_{j}}{\to }\emptyset \;where\;r_{j}=R_{j,basal}C+R_{j}X_{j}$$

Considering these two types of biomolecular reactions, we could write a differential equation for the dynamics of c-di-GMP inside the cell as a function of the detected stimuli. This equation considered *q* proteins of the DGC kind and *l* proteins of the PDE kind, such that *q* + *l* = *m*:

$$\frac{dC}{dt}=\sum_{i=1}^{q}\left(R_{i,basal}+R_{i}X_{i}\right)-\sum_{j=1}^{l}\left(R_{j,basal}C+R_{j}X_{j}\right)$$ [Eq 1]

Then, we used the common steady-state approximation (*dC*/*dt* ~ 0) which assumes that the intracellular levels of c-di-GMP stabilize rapidly after sensing new stimuli. This approximation allowed us to write the following mass-balance equation relating the “basal decay”, “basal synthesis” and the “net responsive” rates:

$$\left(\underset{Basal\;decay}{\underbrace{\sum_{j=1}^{l}R_{j,basal}}}\right)\;C=\underset{Basal\;synthesis}{\underbrace{\sum_{i=1}^{q}R_{i,basal}}}+\underset{Net\;responsive}{\underbrace{\sum_{i=1}^{q}R_{i}X_{i}-\sum_{j=1}^{l}R_{j}X_{j}}}$$ [Eq 2]

With a simple variable substitution we arrived at an equation that determines c-di-GMP levels as a function of a vector of all stimuli sensed by the cell, **X** = {*X*_1_,…,*X*_*m*_}:

$$C(X)=\alpha +\sum_{i=1}^{m}\beta_{i}X_{i}$$ [Eq 3]

Where $\alpha \equiv \frac{\sum_{i=1}^{q}R_{i,basal}}{\sum_{j=1}^{l}R_{j,basal}}$, $\beta_{i}\equiv \frac{R_{i}}{\sum_{j=1}^{l}R_{j,basal}}$ if *i* is a DGC and $\beta_{i}\equiv -\frac{R_{i}}{\sum_{j=1}^{l}R_{j,basal}}$ if *i* is a PDE.

Then, inspired by the FleN-FleQ system, we modeled how an effector module would change its activity depending on the c-di-GMP level. The inverse regulation [29] ensures bacteria express either biofilm genes or motility genes. We modeled this process using a single binary output, *Y*, such that when the output is *Y* = 0 the bacterium expresses motility genes and when *Y* = 1 the bacterium expresses biofilm genes. We defined an effector setpoint *C*_*SP*_, which is the c-di-GMP level at which FleN-FleQ switches from expressing motility genes to expressing biofilm genes. As in previous models of bow-tie networks [4] we used a smooth sigmoidal function (the logistic function) for the effector activity. The probability that a cell expresses biofilm genes depends on c-di-CMP relative to the effector setpoint:

$$P(Y=1|X)=\text{logistic}(C(X)-C_{SP})$$ [Eq 4]

Finally, Eq 4 could be re-written with a simple variable change:

$$P(Y=1|X)=\text{logistic}(\beta_{0}+\sum_{i=1}^{m}\beta_{i}X_{i})$$ [Eq 5]

where $\beta_{0}\equiv \frac{\sum_{i=1}^{q}R_{i,basal}}{\sum_{j=1}^{l}R_{j,basal}+R_{d}}-C_{SP}$.

This model explains how the decision to express biofilm or swarming genes could emerge from simple biochemical reactions (**Fig 3B–3D, S5 Fig**). Despite its simplicity, the model can describe sophisticated information processing such as conditional gene expression. For example, a network with just two sensors (*m* = 2), where sensor *i* = 1 senses mechanical contact with surfaces and sensor *i* = 2 senses a chemical attractant, can be tuned to form biofilm only when it senses a surface (*X*_1_ = 1) but not a chemical attractant (*X*_2_ = 0) by having its *β*’s optimized to express biofilm genes when *X*_1_ = 1 and *X*_2_ = 0. Importantly, the model also shows that the network behavior can be robust to changes in its biochemical components. Robustness is an important feature of biochemical networks [38]. In the c-di-GMP network this means that two different bacteria could express the same phenotype in a given environment despite having different intracellular c-di-GMP levels, as long the biochemical components were such that the values of the compounded *β* parameters remained unchanged. The c-di-GMP network of *P*. *aeruginosa* has potentially more than 40 DGC and PDE proteins (**Fig 2D**). This provides many possibilities to integrate different stimuli and regulate biofilm formation or swarming in different environments—a regulatory complexity that explains the phenotypic diversity observed among the 28 clinical isolates.

### Laboratory selection produces network mutants with specialist phenotypes

**Fig 4:**
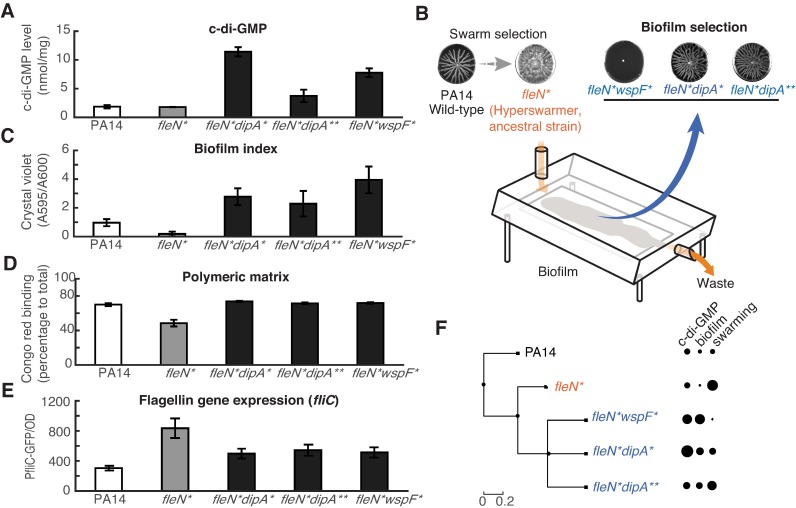
Specialist strains produced by strong selection in laboratory evolution have large-effect alleles in c-di-GMP network. A: Bulk c-di-GMP levels measured for evolved mutants, collected from bacterial colonies. B: Diagram of drip flow biofilm reactor used in biofilm selection. C: Biofilm levels quantified by the crystal violet assay. D: Production of extracellular polymers required for biofilm formation, measured using the Congo-red assay. E: Expression of the gene *fliC* required for flagella synthesis, measured as GFP expressed by the reporter fusion P_*fliC*_-GFP. The data of three evolved mutants fleN*dipA*, fleN*dipA** and fleN*wspF* in B-E are statistically different from ancestral strain fleN* (P<0.05). F: Phylogenetic representation of the mutants evolved in laboratory experiments showing the tradeoff between biofilm and swarming.

The next question is how does selection tune the c-di-GMP network depending on the environments experienced? We first sought out to investigate this question using experimental evolution with the laboratory strain PA14. In the past, we had shown that a swarming environment selected for hyperswarmer mutants with single point mutations in FleN [16]. Here, we analyzed a hyperswarmer mutant from that study—mutant FleN(V178G), from hereon called strain fleN*—to understand whether its phenotype could be explained by our model. The mutant fleN* is a poor biofilm former [16]. Its specialist-swarming phenotype could be either due to having a low level of c-di-GMP or a failure of FleN-FleQ to respond to raising c-di-GMP levels since either possibility could cause the bacterium to stay locked in motility mode. We measured c-di-GMP in fleN* and the levels were the same as in the PA14 wild-type strain (**Fig 4A**). This indicated that the FleN(V178G) mutation decreased the FleN-FleQ response without changing the c-di-GMP level.

To explore whether fleN* could acquire new mutations that recovered its biofilm capabilities, we put this strain under a constant selection for biofilm formation using drip-flow biofilm reactor [39] (**Fig 4B**). After growing biofilms for a few days (**see** methods) we could isolate three distinct mutants of fleN* with recovered biofilm capabilities. Two of these had mutations in the *dipA* gene (DipA^L505R^, DipA^T792P^, called respectively dipA*, dipA**) and one had a mutation in the *wspF* gene (*wspF*^dup776-791^, called wspF*). Interestingly, all three mutants had higher c-di-GMP levels than their fleN* ancestor (**Fig 4A**). We also confirmed—using the Congo red binding assay—that those three mutants indeed decreased their production of extracellular polymers needed for biofilm formation (**Fig 4D**). To summarize, all mutants had decreased swarming (a mild decrease in dipA*, dipA** and a total loss in wspF*, **Fig 4B**), higher c-di-GMP levels than both the wild-type and the fleN* (**Fig 4A**), lower expression of flagella genes (**Fig 4E**), higher surface attachment (**Fig 4C**), and higher production of extracellular matrix (**Fig 4D**). We cloned the dipA*, dipA** and wspF* mutations into the fleN* background and confirmed that these mutations were sufficient to increase capacity for biofilm formation and reduce swarming (**S6 Fig**). Clean deletions (Δ*dipA* and Δ*wspF*) caused similar changes towards more biofilm and less swarming in both the fleN* and wild-type background, indicating (i) that the mutations phenocopied loss-of-function and (ii) that they could work even in the absence of the fleN* mutation (**Fig A,B,C in S7 Fig**).

The raised levels of c-di-GMP suggested that the mutations in dipA*, dipA** and wspF* could be compensating for the decreased sensitivity of FleN-FleQ and allowing the bacteria to recover their biofilm formation. The two proteins encoded by the mutated genes—DipA and WspF—are however functionally very different. DipA has both a GGDEF and a EAL domain and its loss-of-function can increase biofilm formation and decrease biofilm dispersal [40]; results from a screen suggest that DipA acts as a PDE [41]. WspF does not interact with c-di-GMP directly but does so indirectly; it is a methyltransferase that de-methylates the transmembrane Wsp complex that thereafter activates the c-di-GMP synthase WspR [42]. We created double dipA*wspF* and dipA**wspF* mutants in the fleN* background to determine whether the mutations would conflict with each other (**Fig D in S7 Fig**).

Our evolutionary experiments produced mutants that—unlike the clinical strains—had large differences in c-di-GMP, biofilm and swarming caused by a few alleles of large effect. How does our model explain these observations? The laboratory strain PA14 is a generalist capable of both biofilm and swarming. Our model says that the interplay between the c-di-GMP level *C* and the FleN-FleQ setpoint *C*_*SP*_ determines the decision to switch the phenotype. In an environment that favors motility—such as a swarming plate—c-di-GMP would stay below the effector setpoint such that *C* < *C*_*SP*_. In an environment that favors biofilm formation—such as a solid surface—c-di-GMP would raise above the setpoint such that *C* > *C*_*SP*_ (**Fig 3B**). The fleN* hyperswarmer is a swarming specialist that forms weak biofilms despite having the same c-di-GMP level as the wild-type PA14. According to our model, the hyperswarmer has a higher setpoint, *C*_*SP*_′, which would lock the bacteria in motile-mode even when c-di-GMP levels raise to levels *C*_*SP*_′ > *C* > *C*_*SP*_ (**Fig 3C**). The three distinct biofilm-recovery mutants dipA*, dipA** and wspF* could compensate for a higher setpoint by producing more c-di-GMP and raising its level to *C*′ > *C*_*SP*_′. Interestingly, the mutations dipA* and dipA** had milder phenotypes than wspF*; those strains where still capable of both biofilm and swarming despite having higher c-di-GMP levels, whereas wspF* lost its swarming entirely (**Fig 3D**). This suggests that the two *dipA* mutants adjusted their c-di-GMP level to regain their generalist behavior, while the *wspF* mutant became a biofilm specialist (**S5 Fig**).

### The c-di-GMP network as a machine learning classifier

The mutants evolved in the laboratory experienced strong selective pressures, and their phenotypes—caused by large-effect alleles—showed strong associations: biofilm and swarming were anti-correlated (**Fig 4F**, **S8 Fig**). The clinical strains showed weak phenotype associations and only small-effect alleles, suggesting that they had evolved under weak selection. Can our model help unite our clinical and laboratory observations? The link between small-effect alleles and weak selection, well established in evolutionary theory [43], would be difficult to test empirically: the selection experienced by the clinical isolates during their evolution occurred in the past and is now inaccessible to us. We turned to theory to investigate how the strength of selection across fluctuating environments and the architecture of the c-di-GMP network could lead to the diversity of phenotypes seen across the clinical and laboratory strains.

The bow-tie model in Eq 5—which can be derived from biomolecular reaction principles—is mathematically equivalent to the equation for a logistic regression [44], which is a discrete choice model used for classification problems in machine learning. The analogy immediately suggests that the c-di-GMP network may work as a biochemical classifier that integrates many environmental stimuli and classifies to which of the two categories—motility-favoring or biofilm-favoring—a new environment belongs. The network which gives bacteria the ability to change phenotype when they encounter a new environment results from the environmental changes, or fluctuations, experienced during their evolutionary history. Natural selection exerted in each environment works on the bacteria at the population level in a way that resembles telling bacteria—by killing them or letting them live—whether the action was favorable.

How fast the environment changes relatively to the strength at which natural selection acts on the bacterial population is a critical parameter. We call this parameter *n*, the effective length of the evolutionary history. In the extreme case of *n* = 1, selection is so strong that only the last environment matters. A value *n* > 1, but still small, represents a strong selection where the fittest network consistently outperformed its competitors across a small number of environments. The larger the value of *n* the weaker the selection in each environment, and the fittest network is the one that consistently outperformed competitors in a long series of environments.

We derived a mathematical analogy between evolution across fluctuating environments and training a logistic regression classifier to investigate how low *n* (strong selection) can produce specialist networks whereas high *n* (weak selection) favors generalists. Classifiers learn their task by training with large datasets, for example a matrix *m* × *n* of input variables **X** and their correct output **E** = (*E*_1_,…,*E*_*n*_). The likelihood of obtaining the output *Y*_*j*_ = *E*_*j*_ is *P*(*Y*_*j*_ = 1|**X**_***j***_) if *E*_*j*_ = 1, and is 1 − *P*(*Y*_*j*_ = 1|**X**_***j***_) if *E*_*j*_ = 0. This can be written ${P(Y_{j}=1|X_{j})}^{E_{j}}\times {\left(1-P(Y_{j}=1|X_{j})\right)}^{1-E_{j}}$ for brevity. The fitting criterion in a logistic regression is that the values of ***β*** = (*β*_0_,…,*β*_***m***_) should maximize the likelihood of obtaining output **E** from input **X** across the *n* data points:

$$\max_{\beta }\left\{\prod_{j=1}^{n}\pi_{j}^{E_{j}}{\left(1-\pi_{j}\right)}^{\left(1-E_{j}\right)}\right\}\;where\;\pi_{ij}=P(Y_{j}=1|X_{j})=\text{logistic}(\beta_{0}+\sum_{i=1}^{m}\beta_{i}X_{i}^{j}X_{i})$$ [Eq 6]

Evolution across fluctuating environments may be described in a similar way. In our case, each environment *j* ∈ {1,…,*n*} is either a motility-favoring environment, *E*_*j*_ = 0, or a biofilm-favoring environment, *E*_*j*_ = 1, and the fitness *f*_*j*_ in each environment is the agreement between the phenotype favored *E*_*j*_ and the expressed phenotype *Y*_*j*_:

$$f_{j}=\pi_{j}^{E_{j}}{\left(1-\pi_{j}\right)}^{\left(1-E_{j}\right)}\;where\;\pi_{ij}=P(Y_{j}=1|X_{j})$$ [Eq 7]

A classical result from evolutionary theory states that when a diverse population experiences a series of *n* fluctuating environments natural selection will favor the variant with the highest fitness geometric mean across the *n* environments [45]:

$$F=\sqrt[{n}]{\prod_{j=1}^{n}f_{j}}$$ [Eq 8]

Under these conditions, the fittest network across *n* environments would be the one that made best use of the array of *m* stimuli sensed in each environment, **X**_*j*_ = (**X**_*j*1_,…,**X**_*jm*_), and expressed—to the extent possible—the right phenotype. This network is the one with ***β*** = (*β*_0_,…,*β*_***m***_) that maximize geometric mean fitness across the *n* environments:

$$\max_{\beta }\left\{\sqrt[{n}]{\prod_{j=1}^{n}{P(Y_{j}=1|X_{j})}^{E_{j}}\times {\left(1-P(Y_{j}=1|X_{j})\right)}^{1-E_{j}}}\right\}$$ [Eq 9]

which is the same as the criterion for logistic regression, because maximizing the *n*th-root of a quantity is the same as maximizing the quantity itself.

To summarize the analogy, a classical result of evolutionary theory [45] allowed us to conclude that the total set of *m* stimuli sensed during network evolution across *n* fluctuating environments corresponds to a *m* × *n* input matrix, $X=(X_{1}^{T},\ldots ,X_{n}^{T})$, and the phenotypes favored by each of those *n* environments correspond to an output vector, **E** = (*E*_1_,…,*E*_*n*_)^T^. The solution of Eq 6 and Eq 9—the set of values ***β*** that maximizes the quantities described—is the same and so natural selection across fluctuating environments is mathematically equivalent to training a machine learning classifier.

### Strong selection favors specialists and weak selection favors generalists

The analogy above opens the way to investigate how the size of the *m* × *n* matrix determines the fitness of a network in future environments, since it is well known in statistical learning that the size of training data determines the performance of a classifier when it encounters new input data. We carried out simulations where we considered a simple scenario: fluctuating environments that selected for either biofilm or motility, and that occurred with the same probability.

We generated the binary vectors of length *n* to represent the phenotype **E** favored in each environment (*E*_*j*_ = 0 representing swarming selection and *E*_*j*_ = 1 representing biofilm selection) and we created *n* × *m* matrices of noiseless stimuli **X** (*X*_*ij*_ = 0 in a environment favoring swarming and *X*_*ij*_ = 1 in an environment favoring biofilm) and then we swapped the values for a fraction 1 − *η* to add unbiased noise to the stimuli (**supporting material**). We then derived the analytical solution for the best network in the limit of very long evolutionary histories (*n* → ∞) as a function of the signal quality, *η*. This theoretical best network was—by definition—unbiased for biofilm or swarming since the two phenotypes were set to be equally probable. This means that the sensor activities, *β*_1_,…,*β*_*m*_, should all be equal (all stimuli are equally informative and should have the same weight on the network’s response), and their values should increase (the sensors should become more sensitive) with increasing signal quality *η*.

**Fig 5:**
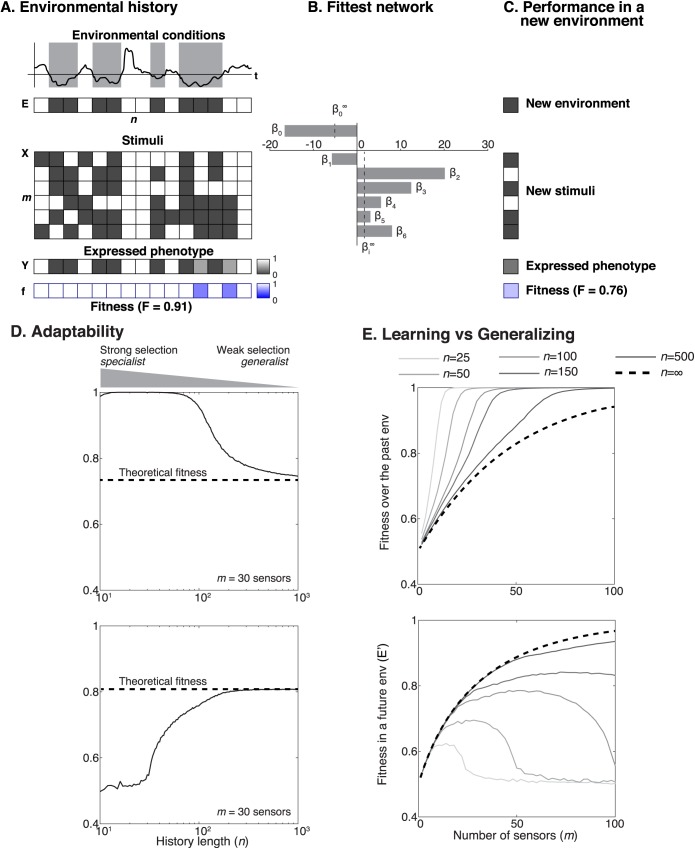
Mathematical model reveals how the c-di-GMP network fitness depends on the strength of selection and on the number of sensors. A: The environment history was modeled as a succession of *n* binary environments *E*, 0-black or 1-white, corresponding respectively to motility- or biofilm-favoring environments. Stimuli (*X*) were generated from each environment by introducing noise to the original signal; the expressed phenotypes *Y* were calculated from the *β*’s and the matrix *X*; the fitness of the network in each environment is the agreement between the expressed phenotype and the favored phenotype in that environment; the fitness across the *n* environments is the geometric mean fitness. B: The fittest network was calculated using logistic regression algorithm. $\beta_{0}^{\infty }$ and $\beta_{i}^{\infty }$ are the fitting parameters of the unbiased network for infinite history. C: The fittest network was presented to a new environment and a new set of stimuli and we calculated the expressed phenotype, as well as the fitness in that new environment. D: The fitness in changing environments depended strongly on *n*, the number of environments that tuned the c-di-GMP network during strain evolution. Strong selection selected for networks adapted to recent environment (small *n*) favoring specialists; weak selection provided the opportunity to learn from a long history of fluctuating environments (large *n*), favoring generalists. E: The fitness achieved by a c-di-GMP network depends on the number of sensory modules (*m*) and the length of evolutionary history (*n*, where small *n* corresponds to strong selection and large *n* corresponds to weak selection). Networks with too many sensors (*m* > *n*/2) performed well in the past but poorly in the future. The curves presented in D-E were obtained from numerical simulations of the scheme described in A-B-C (Logistic regression over a *m* × *n* matrix followed by the estimation of the fitness of the network on one new environment; 1000 independent simulations per conditions *m*, *n*; *η* = 0.6). Arithmetic mean was used to average these simulation results.

We then investigated how the strength of selection determined the network by calculating the network selected with finite values of *n* (**Fig 5A**). This network is the solution of fitting a logistic regression (**Fig 5B**). In contrast to the theoretical best, the calculated network was typically biased to either biofilm or swarming (**Fig 5C**). The bias was stronger for small *n* because it was more likely that the vector of evolutionary histories **E** with small length *n* had an overrepresentation of either biofilm or swarming. We then saw that the stronger the network bias was, the worse the fitness in future environments, **E**′, would be (**Fig 5D**). This result, while expected from statistical learning, has biological insight: it explains that strong selection, such as in our laboratory experiments, can select for specialist networks biased for biofilm or swarming. Weak selection, more likely outside the laboratory, reduces network bias and produces generalists.

We then investigated how the number of sensors in the network, *m*, affected fitness (**Fig 5E, top**). We saw—interestingly—that the future performance of a network increased with the number of sensors *m*, peaked at an intermediate value *m* ~ *n*/2, and then decreased for *m* > *n*/2 (**Fig 5E, bottom**). A network with *m* > *n*/2 had too many components and could be tuned to irrelevant features of past environments that were simply due to noise or under sampling, making it incapable of generalizing in future environments. This is related to statistical overfitting, a well-known phenomenon: the more parameters there are in a statistical model, the easier it is to overfit [46,47]. For the c-di-GMP network this means that the optimal number of sensors for a network depends on the strength of selection across fluctuating environments. A network with too few sensors (*m* < *n*/2) cannot be properly tuned and will be disfavored by natural selection. Networks with too many sensors (*m* > *n*/2), on the other hand, can be over-tuned to the past and maladapted for the future. Optimal networks have a number of sensors *m* ~ *n*/2. Consistent with statistical learning, their maximum achievable fitness is limited by the noise in the stimulus (**S9 Fig**) and increases with the size of the training history, *n* (**Fig 5E**).

The analogy between c-di-GMP signaling and a machine learning classifier explains that weak selection favors generalist bacteria; generalists integrate environmental stimuli and decide between biofilm and swarming according to the environmental fluctuations experienced in their evolutionary history. Evolution in strong selection, on the other hand, favors specialists. This is similar to how small data sets tend to produce biased classifiers.

### Exploiting strong selection to find mutations against biofilm specialism

**Fig 6:**
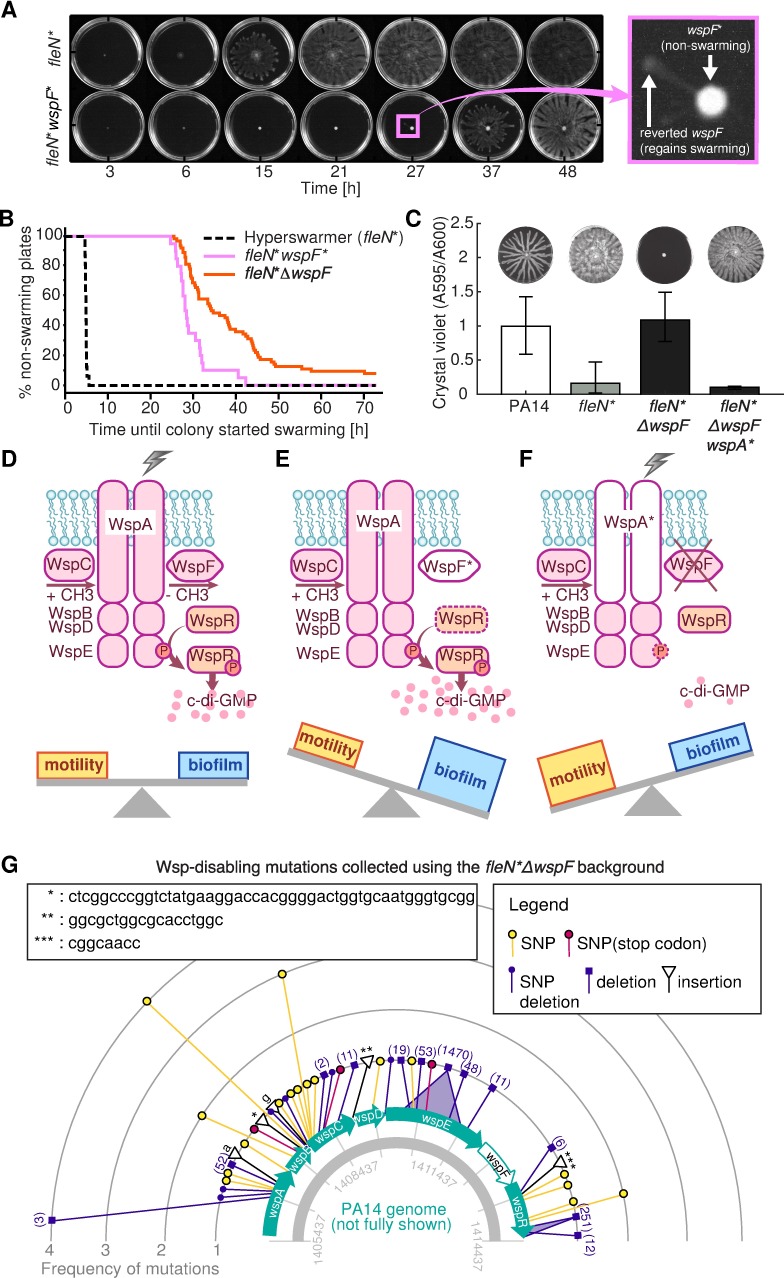
Experimental tests reveal new mutations that regain hyperswarming to a biofilm specialist. A: The wspF* biofilm specialist which has a repeat-insert in the *wspF* gene, initially cannot swarm but regains swarming by losing the repeat-insert when in swarming selection. B: An engineered fleN*Δ*wspF* strain also regains swarming despite lacking the *wspF* gene entirely. Survival analysis reveals that this mutant takes significantly longer than the wspF* to start swarming, but does so eventually. C: A spontaneous mutant in *wspA* regained swarming in the fleN*Δ*wspF* background. D-F: Diagram explaining how Wsp mutations enable switching between extremes of biofilm and swarming. When WspA senses an attachment signal, it transduces the signal to other Wsp proteins that phosphorylate protein WspR, which then produces c-di-GMP and the cells form biofilm (D). When WspF gains the insertion mutation, it fails to demethylate. WspR therefore is hyper-phosphorylated even in the absence of an attachment signal. (E). A Δ*wspF* mutant phenocopies wspF*. However, a spontaneous mutation in *wspA* enables cell to swarm. This mutation impairs biofilm formation even when the cells are placed under biofilm forming condition (F). G: Compilation of mutations identified from the mass swarming selection experiment started with the fleN*Δ*wspF* strain that revealed 43 new Wsp-disabling mutations.

In the light of our model, we sought to exploit strong selection in laboratory environments to search for genetic alterations that might bias the c-di-GMP network towards swarming motility. According to our model, mutations that improve swarming should impact biofilm formation, and could be potentially used as targets against *P*. *aeruginosa* virulence [11]. We first noted that the wspF* strain, a biofilm specialist, had a 16 base-pair insert-repeat which functioned as a reversible DNA switch [48]. This strain, when placed under strong swarming selection for longer than 24 h, generated swarming plumes made of mutants that spontaneously lost the insert (**Fig 6A, S1 Movie**). We repeated this swarming-plume assay with a fleN*Δ*wspF* strain—a biofilm-specialist that lacked the *wspF* gene entirely—to search for mutations that could occur elsewhere and cause the phenotype to switch from biofilm specialism back to swarming. The fleN*Δ*wspF* strain also generated swarming plumes when placed under swarming selection for longer than 24 h (**S2 Movie**), although this took longer than for the wspF* (**Fig 6B**; logrank test P = 0.03). Whole-genome sequencing of one plume isolate revealed a 3 bp deletion in the gene *wspA*(Δ857–859). This fleN*ΔwspFwspA* mutant restored the fleN* phenotype of low biofilm and hyperswarming (**Fig 6C**). WspA is a critical component of the surface-sensing Wsp complex (**Fig 6D–6F**); the switch from biofilm specialism to swarming specialism could be due to an inability of raising c-di-GMP when the bacteria touched a surface.

Having found this one Wsp-disabling mutation, we asked whether strong swarming selection applied to the fleN*Δ*wspF* strain could reveal new Wsp-disabling mutations every time. We repeated swarming-plume experiment 89 times and we used high-throughput sequencing to target-sequence the *wspABCDER* operon of plume isolates. We identified 43 new distinct mutations affecting the Wsp system: 17 deletions, 5 insertions and 21 single nucleotide variants; some of these mutations occurred multiple times (**Fig 6G, Table 1 in S1 Text**). All mutations caused the biofilm specialist to regain its swarming, and are therefore potential targets against *P*. *aeruginosa* biofilm formation.

Interestingly, two plume isolates apparently had no mutations in *wspABCDER*. We sequenced their whole genomes to search for mutations elsewhere. Both mutants had point mutations in another predicted c-di-GMP network gene, PA14_03720 (mutations D378G and E506A). This gene has a GGDEF motif but, intriguingly, a previous study had not detected an effect in biofilm or swarming in a ΔPA14_03720 mutant [41]. The point mutations that we identified in PA14_03720 thus provide an unexpected way to impact the c-di-GMP network and cause loss of biofilm specialism.

## Discussion

We presented empirical results and a new mathematical model that provides a new interpretation of the ubiquitous c-di-GMP network of bacteria that computes like a biochemical machine learning classifier. Our analysis of 28 *P*. *aeruginosa* clinical isolates revealed diverse levels of c-di-GMP, biofilm formation and swarming motility. The three traits were uncorrelated, and the apparent lack of associations seemed to contradict a well-known dichotomy between biofilm and swarming. Phylogenetic analysis showed evidence of a tradeoff, but only among a few closely related strains.

Explaining a significant fraction of the diversity in c-di-GMP, biofilm and swarming seen in our clinical isolates required many small-effect alleles (**Fig 2E**). In contrast, mutants evolved in strong-selecting laboratory conditions had large-effect mutations that caused switches from swarming specialism to biofilm specialism and back (**Figs 4 and 6**). Our mathematical model explains these mutations: Altering the input/output mapping of the c-di-GMP network can lock the bacteria in either biofilm or swarming mode.

We use a classical insight from evolutionary theory—that natural selection across a series of fluctuating environments favors strategies that maximize the geometric mean fitness [45]—to investigate why strains evolved under weak selection (most likely outside the laboratory) have small-effect alleles, whereas strains evolved under strong selection (as we applied in our laboratory evolutionary experiments) have large-effect mutations. We derived a mathematical equivalence between natural selection and training a logistic regression model. This analogy is based on simplifying assumptions and is valid only when the genetic variance within the population is large; in that case selection can choose from wide range of variants and pick the best one. When genetic diversity within the population is low, evolution should resemble reinforcement learning—another learning paradigm, where data is fed online. Mutations in bacteria would correspond to “suggesting” an action, and the environment would “inform” the population whether the action was favorable by killing bacteria or letting them live. Nonetheless, the simplifying assumptions allowed us to investigate the networks with maximum geometric mean fitness and gain biological intuition on the evolution of c-di-GMP. We saw that the strength of selection determines the optimal number of input sensors (**Fig 5E**). Our simulations also explained why networks evolved in strong selection are more likely to be biased—specialists in either biofilm or swarming. These insights helped us unify our clinical and laboratory observations.

The architecture of biochemical networks determines their function [49]. The bow-tie architecture of c-di-GMP suggests a machine learning classifier whose function is to determine, from a set of stimuli, to which of two categories an environment belongs—biofilm-favoring or motility-favoring. It is likely that some of the stimuli sensed by the c-di-GMP network will be redundant; in that case their integration would improve decision-making by averaging out noise [1]. Some stimuli, however, may be complementary; in that case their integration could enable conditional decision-making. Some of those stimuli may help bacteria determine who their neighbors are to better resist cheating—a constant threat to the stability of social behaviors, including biofilm and swarming [50]. Signal integration in a bow-tie network has therefore many advantages. The reliance on a core molecule, however, has a well-known disadvantage [3]: mutations that improve one output can impair the other output(s). Microbiologists had already noted this phenomenon [29]. The tradeoff also occurred in our experimentally evolved hyperswarmers, which lacked biofilm formation [16]. We saw it again here in the *dipA* and *wspF* mutants (**Fig 4F, S8 Fig**) which improved biofilm formation but decreased swarming. And we leveraged the tradeoff in the plume-isolation assay to find 45 new mutations that caused loss of biofilm specialism (**Fig 6**).

Our network model—simple on purpose—made several notable assumptions. First, the model assumed deterministic and steady-state biochemical reactions. The model also assumed one single c-di-GMP pool within the cell; some evidence suggests there may be many pools [51] although this is under debate [52]. Our goal, however, was to demonstrate that even a simple biochemical network could compute like a machine learning classifier. Including dynamics, stochasticity and more hidden nodes in the c-di-GMP network could add even more sophisticated computation (**S10 Fig**) and the network could eventually approach the performance of a deep neural network [4]. Understanding the function and evolution of such biochemical networks is where the concepts of machine learning—already a powerful tool to interpret complex biological data [53]—could help elucidate the evolution of biological systems [54].

Our results shed light on bacterial evolution in three important ways: First, they provide a mechanism of adaptation on a range of timescales, from the second to minutes involved in the swarm/biofilm decision to the timescales involved in evolution.

Second, they suggest that we may be able to estimate the evolutionary history—the number of environments that a bacterium has experienced in its evolution—from the number of sensors in a network. Our model says that well-adapted networks should have a number of sensors (*m*) that is proportional to the evolutionary history (*n*). In our simplified model, this relationship is *m* = *n*/2. If we know more about the stimuli and dynamics of a biochemical network such as c-di-GMP in *P*. *aeruginosa* (*m* ~ = 53), we should be able to calculate the effective size of the evolutionary history that *P*. *aeruginosa* has experienced. This analysis could be made across different species to compare their evolutionary histories and perhaps even predict future fitness.

Third, the idea of “overfitting” to past experiences suggests network weakness that we could exploit. For this application, it will be important to know when is the environment change “extremely rapid” versus “not rapid enough”. The conventional view is that most natural environments change slowly most of the time, as natural environments tend to be smooth, punctuated by rare but large change. Many laboratory settings are “not rapid enough” as well. For example, the drip flow biofilm experiments shown here were “not rapid enough” for all cells to wash away; this was on purpose so we could obtain mutants that recovered biofilm formation. The hygienic environments in hospitals are often “not rapid enough” either, and bacteria can adapt and become resistant to antibiotics. We may already be familiar with the “overfitting” idea: Almost all of our methods to kill bacteria come from knowing that bacteria “overfit” what they experienced in the past, and we need to artificially change the environment fast, such as in a sudden rise in antibiotic concentration or ultraviolet radiation, to effectively kill bacteria. We could take advantage of new knowledge to engineer combinations of environmental stimuli that bacteria never encountered before and trigger a maladapted response—for example biofilm dispersal—in a way that treats infection but prevents resistance.

## Methods

See supplementary materials for additional methods details.

### Strains and culture conditions

All strains were grown overnight in lysogeny broth (LB) at 37°C with shaking at 250 rpm. Swarming media consisted of 0.5% agar (Bacto) supplemented with 5g/L casamino acid, 1 mM MgSO_4_, 0.1 mM CaCl_2_ and 1X buffer (12 g/L Na_2_HPO_4_ (Fisher Scientific), 15 g/L KH_2_PO_4_ (Fisher Scientific) and 2.5 g/L NaCl, pH6.7) [55]. Biofilm assays were carried out in 96-well plates in 1% trypton at 25°C for 24 hours and quantified by crystal violet staining [56]. c-di-GMP measurements were obtained from colony biofilms incubated on trypton plates with 1% agar.

### Whole-genome sequencing, annotation and mutation identification

The *P*. *aeruginosa* clinical isolates were sequenced using PacBio by the Genomics Facility at the Icahn School of Medicine at Mount Sinai (Robert Serba, PI), the genomes were annotated by the PATRIC [57] and the LASSO regression was done with glmnet [58]. Isogenic clones of PA14 were sequenced using Illumina MiSeq platform and mutations were identified using breseq [59].

### Mathematical modeling and data analysis

All data analysis and plotting was conducted in Matlab, except for the Moran test for phylogenetic signal determination conducted in R using package ‘adephylo’ [60]. Mathematical model was implemented in Matlab based on the logistic regression in function mnrfit.m.

## Supporting information

S1 Fig **Correlation of biofilm and swarming against c-di-GMP in clinical isolates (A, B).** The linear fit and coefficient (R) are shown in each plot.
(EPS)

S2 Fig Correlation between biofilm and swarming in clinical isolates. A: raw correlation. B: removing phylogeny dependence using phylogenetic generalized least square regression. C: phylogeny dependence removed, excluding strains M55212, F30658 (PAO1 clade) and M37351 (PA14 clade) that contributed most to the correlation in B.
(EPS)

S3 Fig Moran’s I tests for c-di-GMP, biofilm and swarming. Only c-di-GMP shows significant phylogenetic signal (p = 0.006).
(EPS)

S4 Fig Closely related clinical isolates show anti-correlation between biofilm and swarming after phylogenetic correction, but only when they have different phenotypes. We highlight three groups of strains that had closely related genomes (see groups highlighted in **Fig 2D**) after phylogenetic generalized least square regression. Swarming and biofilm show strong anti-correlations in first two groups (A,B), which indicates a tradeoff between these two phenotypes. No correlation is seen in the third group (C), because strains in this group have very similar phenotypes to each other. The tradeoff was less detectable across the entire phylogenetic tree since the whole tree includes phylogenetically distant strains (see main text).
(TIF)

S5 Fig Diverse levels of c-di-GMP, biofilm and motility among the laboratory-evolved PA14 mutants interpreted in the light of the bow-tie model. The phenotypes of *P*. *aeruginosa* PA14 (A) and its fleN*, wspF* and dipA* mutants can be interpreted according to changes in the effector module (*fleN*) and a sensory module (*wspF* or *dipA*). The x-axis represents the intracellular c-di-GMP level and the y-axis represents the phenotypic response (network output). B: The *fleN* mutation changes the setpoint for c-di-GMP response locking the hyperflagellated *fleN*-mutated hyperswarmer in motility mode. C: Mutations in *dipA* increase c-di-GMP more moderately than those in *wspF*; the *dipA* mutation in a wild-type *fleN* background causes biofilm specialism. D: The milder effect of the *dipA* mutation allows the *fleN*-mutated background to remain a generalist despite a higher c-di-GMP level compared to the PA14 wild-type. E: Mutations in *wspF* increase the basal production of c-di-GMP, shifting the c-di-GMP dynamic range upward to lock the bacteria in biofilm mode. F: The *wspF* mutation effect is strong and locks bacteria in biofilm mode even in the *fleN* mutated background.
(EPS)

S6 Fig Removing the *dipA* and *wspF* mutations restore ancestral phenotypes: Increasing swarming and impairing biofilm formation. (TIFF)

S7 Fig Biofilm-recovery mutations in dipA*, dipA** and wspF* suggest loss-of-function of encoded proteins, causing an increase in biofilm formation even in the absence of the fleN* mutation. A: A clean deletion of *dipA* in the fleN* background phenocopies the dipA* and dipA** mutations by increasing biofilm formation and decreasing swarming relative to the ancestral the fleN* background. B: The dipA* and dipA** mutations increase biofilm and lead to total loss of swarming in the wild-type background. C: The wspF* mutations in the wild-type background have same effect as in the fleN* background; we also show that a spontaneous mutations in *wspR* suppressed the Δ*wspF*. D: The phenotype of *wspF* mutations (wspF* or Δ *wspF*) dominates over the phenotype of dipA*, dipA** in triple *fleN*/*wspF*/*dipA* mutants.
(TIFF)

S8 Fig Isogenic mutants evolved from PA14 show better correlation among three phenotypes: c-di-GMP, biofilm and swarming. (EPS)

S9 Fig A: The fitness achieved by a c-di-GMP network depends on the fidelity *η*. Faithful stimuli (high *η*) require fewer sensors to achieve a given fitness. B: The fitness in future environments also depends on the fidelity *η*. However, the optimal number of sensors (*m* = *n*/2) is independent of *η*. The simulations are repeated 1000 times per conditions and averaged arithmetically, with history length *n* = 50.
(EPS)

S10 Fig An expanded network model with multiple c-di-GMP pools retains the bow-tie architecture. Our model could be expanded to accommodate additional features including sub-cellular compartments with distinct c-di-GMP levels, where the location of effectors respond to local c-di-GMP pool and regulate downstream phenotypes. The size of local pool could vary due to the relative location to c-di-GMP synthesis/degradation modules. There also could be positive or negative feedback loops that regulate downstream pathways and leads to one or multiple phenotype changes. For example, the green color feedback affect sensor A would increase/decrease c-di-GMP pool I and II, therefore change both phenotype I and II at the same time. The blue feedback would only regulate B and phenotype III.
(EPS)

S1 Text Supporting materials. Detailed information on phylogenetic generalized least squares (PGLS) method, LASSO analysis, logistic regression of c-di-GMP network, Wsp module mutations identified from swarming selection, and expanded materials and methods. **Table 1.** Mutations identified in the Wsp system from fleN*ΔwspF (see **Fig 6G**)**. Table 2.** Primer sequences used in this study.
(DOCX)

S1 Movie Time lapse of fleN* mutant and fleN*wspF* mutant cells swarming on soft agar petri dish. fleN* cells start to swarm before 6h. fleN*wspF* cells remain in the inoculum. Mutations emerge in fleN*wspF* cells and enable them to swarm after 20h. The time is in hh:mm format.
(MP4)

S2 Movie Time lapse of fleN* and fleN*ΔwspF mutant cells swarming on soft agar petri dish. fleN* cells start to swarm before 6h. Mutations emerge in fleN*ΔwspF strain and enable the cells to swarm after 30h. The time is in hh:mm format.
(MP4)
