# Supplementary figures and images for: Bow-tie signaling in c-di-GMP: Machine learning in a simple biochemical network
Source: PLoS Comput Biol. 2017 Aug 2;13(8):e1005677. doi: 10.1371/journal.pcbi.1005677 (PMC5555705; doi:10.1371/journal.pcbi.1005677)

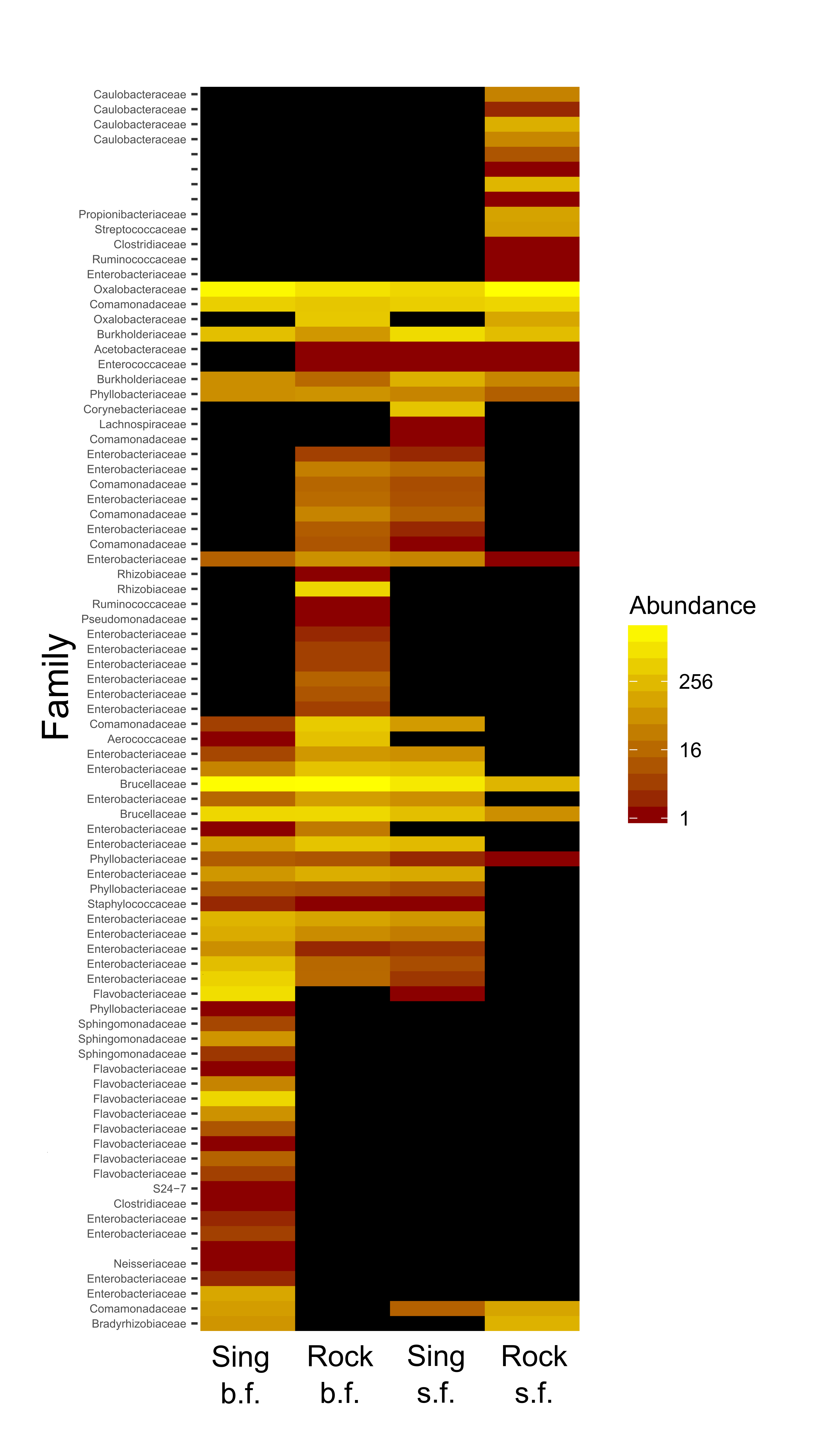

Supplement: S4 Fig — We highlight three groups of strains that had closely related genomes (see groups highlighted in Fig 2D) after phylogenetic generalized least square regression. Swarming and biofilm show strong anti-correlations in first two groups (A,B), which indicates a tradeoff between these two phenotypes. No correlation is seen in the third group (C), because strains in this group have very similar phenotypes to each other. The tradeoff was less detectable across the entire phylogenetic tree since the whole tree includes phylogenetically distant strains (see main text). (TIF) [file pcbi.1005677.s004.tif]

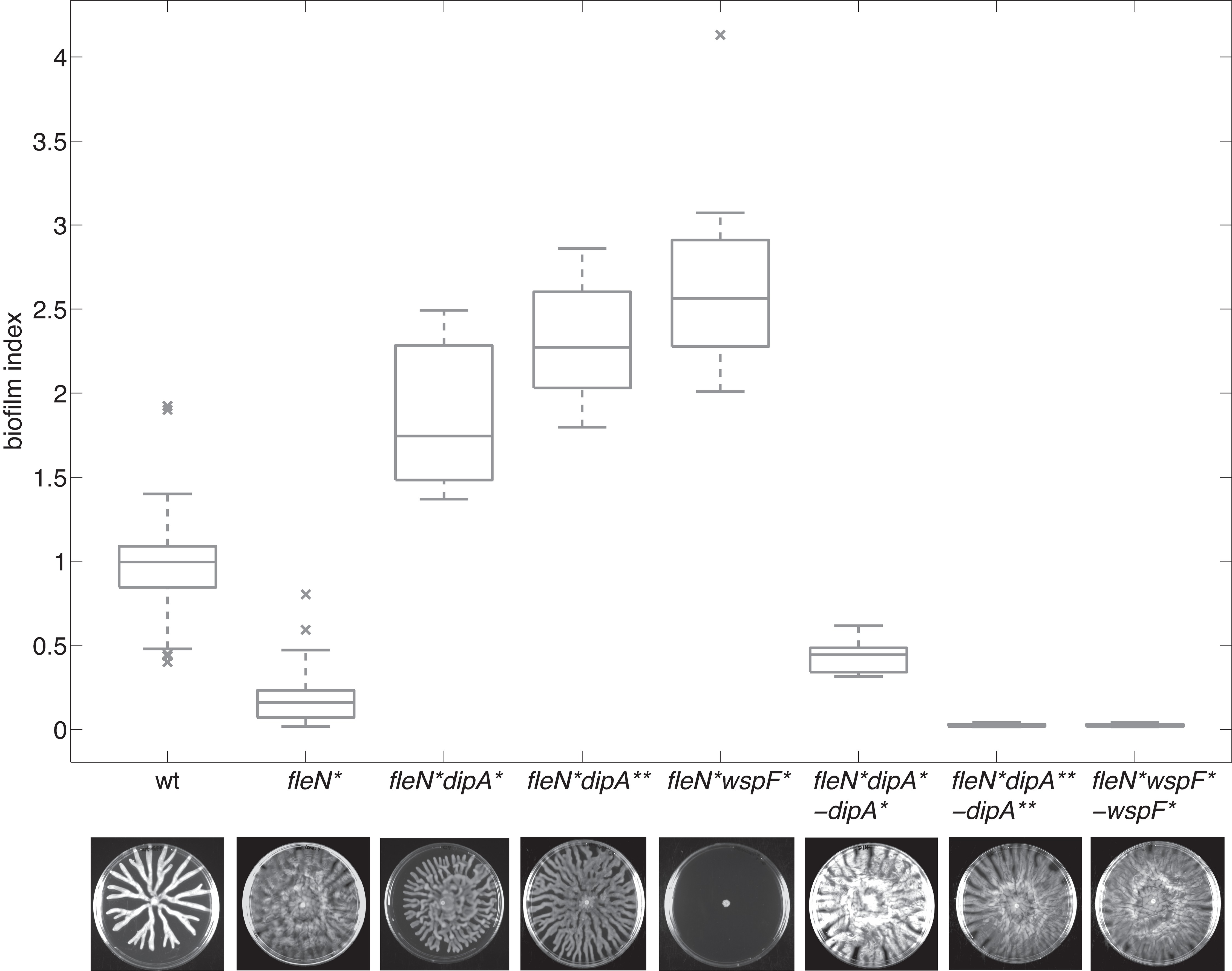

Supplement: S6 Fig — (TIFF) [file pcbi.1005677.s006.tiff]

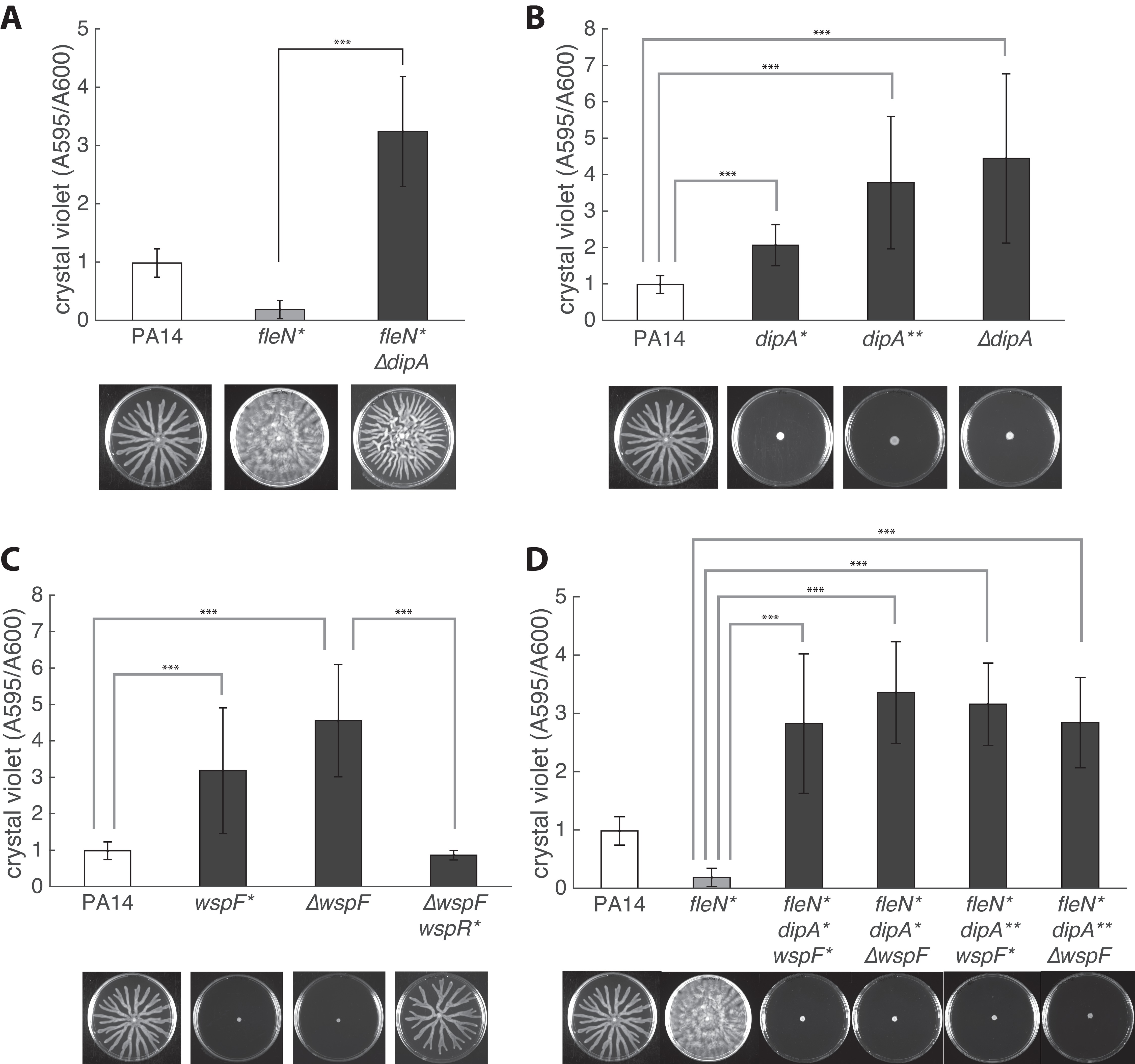

Supplement: S7 Fig — A: A clean deletion of dipA in the fleN* background phenocopies the dipA* and dipA** mutations by increasing biofilm formation and decreasing swarming relative to the ancestral the fleN* background. B: The dipA* and dipA** mutations increase biofilm and lead to total loss of swarming in the wild-type background. C: The wspF* mutations in the wild-type background have same effect as in the fleN* background; we also show that a spontaneous mutations in wspR suppressed the ΔwspF. D: The phenotype of wspF mutations (wspF* or Δ wspF) dominates over the phenotype of dipA*, dipA** in triple fleN/wspF/dipA mutants. (TIFF) [file pcbi.1005677.s007.tiff]
