# Supplementary material for: Bow-tie signaling in c-di-GMP: Machine learning in a simple biochemical network
Source: PLoS Comput Biol. 2017 Aug 2;13(8):e1005677. doi: 10.1371/journal.pcbi.1005677 (PMC5555705; doi:10.1371/journal.pcbi.1005677)
Supplement: S1 Text — Detailed information on phylogenetic generalized least squares (PGLS) method, LASSO analysis, logistic regression of c-di-GMP network, Wsp module mutations identified from swarming selection, and expanded materials and methods. Table 1. Mutations identified in the Wsp system from fleN*ΔwspF (see Fig 6G). Table 2. Primer sequences used in this study. (DOCX) [file pcbi.1005677.s011.docx]

# Supporting material for “Bow-tie model of c-di-GMP: machine learning by natural selection in a ubiquitous biochemical network”

## Content list:

- Phylogenetic generalized least squares and LASSO analysis of phenotypic diversity
- Logistic regression model of c-di-GMP bow-tie network
- Mutants in Wsp module identified from swarming selection
- Expanded Materials and Methods section
  - Phenotypic assays
  - Biofilm selection in drip-flow reactor
  - Construction of mutants by allelic exchange
  - Biofilm colony morphology and c-di-GMP quantification
  - PacBio sequencing and *de novo* assembly of clinical *P. aeruginosa* strains
  - Computational analysis of genomic sequences
- Supplementary references

## Removing phylogenetic dependence in phenotypes using phylogenetic generalized least squares

The analysis of c-di-GMP variants and how they associate with c-di-GMP, biofilm and swarming shown in the main text by removing phylogenetic dependence that is based on the phylogenetic generalized least squares methods (1). To explain the logic behind this method consider the association between biofilm and swarming that we analyzed in **Fig. SF3**. In this example, the phenotype of each strain is defined by two traits, $s$ (swarming motility) and $b$ (biofilm formation), represented by a two-dimensional phenotype vector $\vec{p}=(s, b)$. When a selective pressure, represented by vector $\boldsymbol{\Delta}\vec{g}$, changes the genotypic composition of a population the average phenotype changes according to:

$$\boldsymbol{\Delta}\vec{p}\boldsymbol{=}f\left( \boldsymbol{\Delta}\vec{g} \right)$$

where $f\left( \boldsymbol{\Delta}\vec{g} \right)$ is an arbitrary function that maps genotypic change to phenotype change. The structure of $f\left( \boldsymbol{\Delta}\vec{g} \right)$ is unknown to us, but we known that it can impose constraints on the direction of phenotypic evolution (2, 3). For example, if $s$ and $b$ were strongly constrained by having a mutual regulation then the two traits could follow a linear equation:

$$b\boldsymbol{=}\alpha+\beta s$$

If there was a tradeoff between biofilm and swarming then the slope in this linear equation would be negative, i.e. ${\partial b}/{\partial s}=\beta<0.$ However, the linear association, if it exists, may apply only when $f\left( \boldsymbol{\Delta}\vec{g} \right)$ is constant, which may be valid only when the strains compared have large phenotypic differences but few genetic differences (large $\boldsymbol{\Delta}\vec{p}$ but small $\boldsymbol{\Delta}\vec{g}$). This assumption does not hold for distant strains because they will differ in multiple genetic polymorphisms that can affect the phenotype (4): a tradeoff that is clear in closely related strains, such as isogenic mutants, can be undetectable in distant strains (5).

We can use phylogenetic generalized least squares (1) to account for the phylogenetic distances in addition to random noise. In matricial form the generalized linear model is

$$\mathbf{B}\boldsymbol{=}\alpha+\beta\mathbf{S}+\gamma\boldsymbol{T+\epsilon}$$

where $\mathbf{S}$ and $\mathbf{B}$ are arrays of swarming and biofilm measurements from different strains, $\mathbf{T}$ compensates for phylogenetic distances and $\boldsymbol{\epsilon}$ accounts for the stochasticity such as measurement errors. We computed a phylogenetic variance-covariance matrix $\mathbf{G}$ from the sequenced genomes. Then, assuming that $\boldsymbol{\epsilon}$ is normally distributed, we fit the equation above by solving generalized least square regression:

$$\beta\boldsymbol{=}\left( \mathbf{S}\mathbf{'G}^{-1}\mathbf{S} \right)^{\boldsymbol{-1}}\mathbf{S'}\mathbf{G}^{-1}\mathbf{B}$$

$\mathbf{T}$ is a matrix decomposition of $\mathbf{G}$ by Cholesky decomposition, such that $\mathbf{T}\mathbf{T}^{\mathbf{'}}\mathbf{=G}$. $\beta$ is the slope of an ordinary least square regression between $s$ and $b$ after correcting for phylogeny. In other words:

$$\beta\boldsymbol{=}\left( \mathbf{S}_{\boldsymbol{c}}\mathbf{'}\mathbf{S}_{\boldsymbol{c}} \right)^{\boldsymbol{-1}}\mathbf{S}_{\boldsymbol{c}}\mathbf{'}\boldsymbol{B}_{\boldsymbol{c}}$$

where the trait values corrected for phylogeny, $\boldsymbol{B}_{\boldsymbol{c}}$ and $\boldsymbol{S}_{\boldsymbol{c}}$, are obtained by a least square regression using the phylogenetic information contained in $\mathbf{T}$:

$$\mathbf{S}_{\boldsymbol{c}}\boldsymbol{=}\left( \mathbf{T'T} \right)^{\boldsymbol{-1}}\mathbf{T'S}$$

$$\mathbf{B}_{\boldsymbol{c}}\boldsymbol{=}\left( \mathbf{T'T} \right)^{\boldsymbol{-1}}\mathbf{T'B}$$

Phylogenetic generalized least squares have not only a statistical interpretation but also a mechanistic interpretation: the method assumes that the traits evolve along the branches of a phylogenetic tree according to an unbiased random walk (6), and determines if their variation is correlated.

To determine the c-di-GMP patterns of genetic variation that explain phenotypic diversity using the *lasso* regression we first computed the phylogenetically corrected values for the c-di-GMP levels:

$$\mathbf{C}_{\boldsymbol{c}}\boldsymbol{=}\left( \mathbf{T}^{\mathbf{'}}\mathbf{T} \right)^{\boldsymbol{-1}}\mathbf{T}^{\mathbf{'}}$$

The levels of c-di-GMP, biofilm and swarming was logistically transformed and centered using z-score. The outcome matrix make up a 3-dimensional phenotype variable $\mathbf{Y=}\left[ \mathbf{C}_{\boldsymbol{c}}\boldsymbol{,}\mathbf{B}_{\boldsymbol{c}}\boldsymbol{,}\mathbf{S}_{\boldsymbol{c}} \right]\boldsymbol{.}$ Then, we identified the presence and absence patterns for genetic polymorphisms in putative c-di-GMP network genes (the number of network genes found in each strain is listed in **Fig. 2D**) and normalized each variant pattern using z-score technique; we corrected these presence-absence patterns for phylogeny and called it the covariate matrix $\mathbf{X}$. Finally, we used the *lasso* method (7) to determine the matrix of coefficients $\boldsymbol{\beta}$ that associate genetic variation in c-di-GMP genes ($\mathbf{X}$) to the phenotypic variation ($\mathbf{Y}$) by minimizing the quantity:

$$\left\{ \frac{1}{N}\left\| \mathbf{Y}-\mathbf{X}\boldsymbol{\beta} \right\|_{2}^{2}+\lambda\left\| \boldsymbol{\beta} \right\|_{1} \right\}$$

Where $N$ is the number of datapoints in our analysis (the number of strains). The *lasso* uses the penalty parameter $\lambda$ to force the coefficients (entries in matrix $\boldsymbol{\beta}$) to go to zero: The higher the value of $\lambda$, the less parameters a model will have. To determined the number of c-di-GMP genetic variants required to explain a certain percentage of the phenotypic deviance we simply varied the value of $\lambda$ from $\lambda_{min}=e^{-10}$ to $\lambda_{max}=e^{8}$, and determined (i) the number of non-zero rows in the matrix of $\boldsymbol{\beta}$ and (ii) the phenotypic deviance explained at each value of $\lambda$ (**Fig. 2E’**).

Note that the goal was not to determine which genes affect the phenotype. We had two goals for the *lasso* analysis: First, we aimed to determine how many genetic variants would be needed to explain the phenotypic diversity in a best case scenario; we fulfilled this goal by determining that explaining 63% (95% of the maximum DE explained by variants in c-di-GMP network) of the deviance required at least 18 variants (**Fig. 3E**). Second, we aimed to determine whether genetic associations with phenotype were strong or weak; we found that the best of the 18 variants could only explain 23.3% of the deviance when considered on its own (**Fig. 3F**). The *lasso* technique provides interpretable models and not necessarily the only model—for example when two columns in matrix $\mathbf{X}$ co-vary, the *lasso* will pick one column and discard the other (7). Thus, the *lasso* provides a minimal model. The correct pattern of genotypic association is likely more complex, and the effect of genetic variants on c-di-GMP, biofilm and swarming are likely to be even smaller than the ones determined by the model with 18 variants.

## Logistic regression model of c-di-GMP bow-tie network

In our model we assumed for simplicity that each stimulus $X_{i}$ was binary: a true stimulus $X_{i}=0$ signaled an environment favoring motility and a true stimulus $X_{i}=1$ signaled an environment favoring biofilm. Stimuli experienced by bacteria can be noisy (8) and their signal untrue; we used parameter η, which we called signal fidelity, to model the probability that a stimulus reported true information. A stimulus $X_{i}=1$, regardless of whether it was true or not, activated the sensory module $i$ to produce (in the case of a DGC) or degrade (in the case of a PDE) c-di-GMP. We modeled the response of each sensory module $i$ with a basal rate $\alpha_{i}$ and a response factor $\beta_{i}$ such that the contribution to the c-di-GMP pool was the result of $\alpha_{i}+\beta_{i}X_{i}$. The sum of all contributions, $\sum_{i=1}^{m} \left( \alpha_{i}+\beta_{i}X_{i} \right)$, represented the steady-state intracellular c-di-GMP levels in response to stimulus vector $\mathbf{X}$.

To explain the general model, consider the case of a basic building block: a single effector module that regulates expression of motility or biofilm genes. The model represents the FleQ-FleN effector complex that responds to increasing c-di-GMP levels by repressing flagella synthesis and upregulating biofilm matrix (9, 10), and therefore governs the switch from motile to biofilm mode. In this model, parameter $\alpha_{0}$ represented the effector’s setpoint: c-di-GMP levels must be higher than $\alpha_{0}$ to switch from motility to biofilm. The sum $-\alpha_{0}+\sum_{i=1}^{m} \left( \alpha_{i}+\beta_{i}X_{i} \right)$ determined the phenotypic output which we represented as a variable $Y$. When $Y=0$ the cell expressed motility genes and when $Y=1$ the cell expressed biofilm genes. As in a previous model of bow-tie networking (11) we used a smooth sigmoidal function, the logistic function, to represent the conversion of c-di-GMP to output, which in our case gave the probability of expressing biofilm genes:

$P\left( Y=1|\mathbf{X} \right)=\text{logistic}\left( \beta_{0}+\sum_{i=1}^{m} \beta_{i}X_{i} \right)$ [derived from eq. 2 from main text]

Here, $\beta_{0}\equiv{-\alpha}_{0}+\sum_{i=1}^{m} \alpha_{i}$, represented the difference between the effector setpoint and the basal level of c-di-GMP.

To analyze how the environments sampled during evolution determined future network performance. We considered only a single phenotype per environment and not bet-hedging strategies that would generate phenotypic variants (12). Each past environment $E_{j}$ presented $m$ stimuli that could be sensed by the network. We determined the fittest possible network produced by a series of $n$ environments by calculating the values of $\beta_{0},\ldots,\beta_{m}$ that maximized the matching between the phenotypic output favored in that environment, $Y_{j}$, and the array of stimuli presented by the environment, $X_{ij}$ (where $i=1,\ldots,m$ and $j=1,\ldots,n$). The fittest network, i.e. the optimal values **β**=($\beta_{0}$, …, $\beta_{m}$) where obtained by fitting a logistic regression model to find the best association between the $n\times m$ matrix $\mathbf{X}$ and a $n\times1$ array $\mathbf{Y}$. Logistic regression is a classification algorithm often used in the field of machine learning (13). Natural selection is an incremental process where the best theoretical network may be impossible to reach due to mechanistic contingencies. The goal of logistic regression is not to simulate the process of natural selection but rather to calculate its asymptotic result—the best network for a given evolutionary history. Logistic regression allowed us to focus on how the quality of the information experienced in the past determined future network performance, irrespective of contingencies.

### Analysis of the logistic regression

The rules for our bow-tie model simulations are the following:

- The environment ($E$) can either favor motility ($E=0$) or biofilm formation ($E=1$) with equal probability.
- The $m$ sensors are noisy and may transform $X_{i}=0$ to 1 or $X_{i}=1$ to 0, with probability $1-\eta$ (where $\eta$. is the fidelity of the sensory module)
- The network integrates the stimuli to compute $P\left( Y=1 \right| \{X_{i}\})$

The following diagram illustrates the probabilities of the different environments and stimuli presented by those environments for a two-stimulus case:


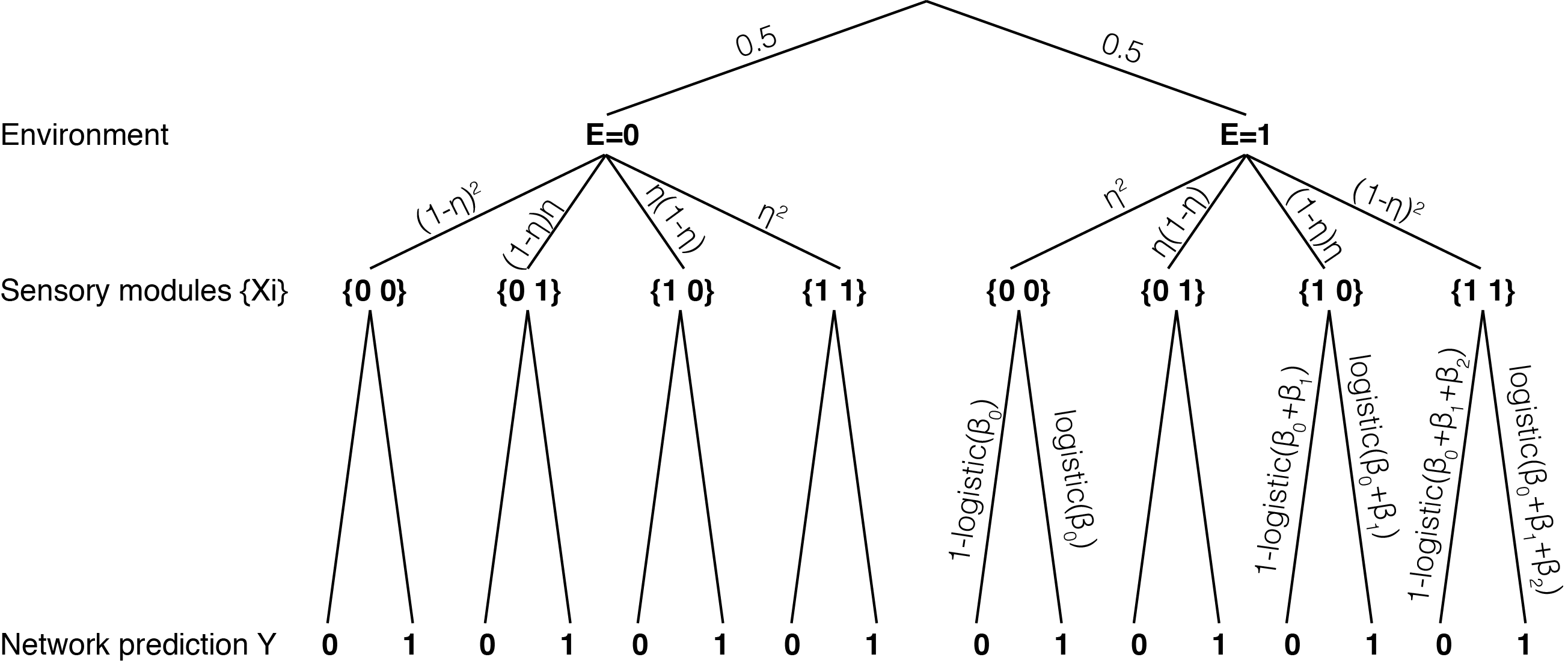


The logistic regression computes the probability of expressing biofilm as

$P\left( Y=1 \right| \{X_{i}\})=Logistic(\beta_{0}+\sum_{i=1}^{m} \beta_{i}X_{i})$ (1)

where $Logistic\left( x \right)= \frac{1}{1+e^{-x}}$. Considering a sequence of *n* independent environments $E_{j}$ and $X_{ij}$ (with $j=1\ldots n$), the set of $\beta_{i}$ that maximizes the joint probability $\prod_{j=1}^{n} P\left( Y_{j} \right| E_{j})$ is given by the logistic regression algorithm, which uses maximum likelihood algorithm. We then obtain the optimal $\beta_{i}$ given the limited length of history and the level of noise in sensory modules.

### Theoretical solution for infinite history length

The logistic regression aims to identify the $\beta_{i}$ values that estimate the best the probability $P\left( Y=1 \right| \{X_{i}\})$. This task improves with the number of environments is greater (more samples). Ultimately, if the number of environments is infinite (infinite sample number), $\beta_{i}$ converge to values that can be calculated analytically.

We aim to calculate the theoretical values of $\beta_{0}$ and $\beta_{i}$ ($i>0$), in the case where:

- subsampling doesn’t affect the logistic regression, i.e. when the history length is infinite.
- all sensory modules are equivalent. Therefore, since history length is infinite, all $\beta_{i}$ (i=1 to m) should be equal: $\beta_{i}= \tilde{\beta}$.
- the environmental series is unbiased: $P\left( Y=1 \right)=P\left( Y=0 \right)=0.5$.

The faithful signal has a probability $1-\eta$ of flipping 0 to 1 or 1 to 0. Therefore

$$P\left( \left\{ X_{i} \right\} \right| E=1)=\prod_{i=1}^{m} {(1-\eta)}^{1-X_{i}}\eta^{X_{i}}$$

Hence

$P\left( \left\{ X_{i} \right\} \right| E=1)={(1-\eta)}^{k}\eta^{m-k}$ (2)

and

$P\left( \left\{ X_{i} \right\} \right| E=0)=\eta^{k}(1-{\eta)}^{m-k}$ (3)

where *k* is the number of 0’s in $\{X_{i}\}$. The theoretical situation for infinite time and perfect sampling (for all (14)) should give $P(E=1 | \left\{ X_{i} \right\})=P(Y=1 \left| \left\{ X_{i} \right\} \right)$, which means that the probability to infer the environment from (14) with the logistic regression is the same as the conditional probability $P(E=1 | \left\{ X_{i} \right\})$. With Bayes’ formula:

$$\frac{P\left( \left\{ X_{i} \right\} \right| E=1)}{P(E=1)}=\frac{P\left( \left\{ X_{i} \right\} \right| Y=1)}{P(Y=1)}$$

Finally,

$P\left( \left\{ X_{i} \right\} \right| E=1)=P\left( \left\{ X_{i} \right\} \right| Y=1)$ (4)

since $P\left( E=1 \right)=P\left( Y=1 \right)=0.5$. Bayes’ formula:

$$P(Y=1 | \left\{ X_{i} \right\})=\frac{P\left( \left\{ X_{i} \right\} \right| Y=1)}{P\left( \left\{ X_{i} \right\} \right)}$$

$P(Y=1 | \left\{ X_{i} \right\})=\frac{P\left( \left\{ X_{i} \right\} \right| Y=1)}{P\left( \left\{ X_{i} \right\} | Y=1 \right)+P\left( \left\{ X_{i} \right\} | Y=0 \right)}$ (5)

With (4) and (5),

$P(Y=1 | \left\{ X_{i} \right\})=\frac{P\left( \left\{ X_{i} \right\} \right| Y=1)}{P\left( \left\{ X_{i} \right\} | E=1 \right)+P\left( \left\{ X_{i} \right\} | E=0 \right)}$ (6)

We study two particular cases:

$\left\{ X_{i} \right\}=\{0 0\ldots0\}$

1. gives $P\left( Y=1 | \left\{ 0 0\ldots0 \right\} \right)=Logistic(\beta_{0})$
2. gives $P\left( \left\{ 0 0\ldots0 \right\} | E=1 \right)={(1-\eta)}^{m}$
3. gives $P\left( \left\{ 0 0\ldots0 \right\} | E=0 \right)=\eta^{m}$

(6) gives $Logistic\left( \beta_{0} \right)= \frac{{(1-\eta)}^{m}}{{(1-\eta)}^{m}+\eta^{m}}$

Finally, $\beta_{0}= -m log(\frac{\eta}{1-\eta})$

$\left\{ X_{i} \right\}=\{1 1\ldots1\}$

1. gives $P\left( Y=1 | \left\{ 1 1\ldots1 \right\} \right)=Logistic(\beta_{0}+m\tilde{\beta})$
2. gives $P\left( \left\{ 1 1\ldots1 \right\} | E=1 \right)=\eta^{m}$
3. gives $P\left( \left\{ 1 1\ldots1 \right\} | E=0 \right)={(1-\eta)}^{m}$

(6) gives $Logistic\left( \beta_{0}+m\tilde{\beta} \right)= \frac{\eta^{m}}{{(1-\eta)}^{m}+\eta^{m}}$

Finally, $\tilde{\beta}= 2 log(\frac{\eta}{1-\eta})$

We obtain the final result:

$$P\left( Y=1 | \left\{ X_{i} \right\} \right)=Logistic(log(\frac{\eta}{1-\eta})\sum_{i=1}^{m} (2X_{i}-1))$$

### Theoretical fitness of the network trained in infinite history

We now aim to calculate the theoretical fitness of the logistic regression, in the case of infinite history length. We define the fitness as the agreement between the expressed phenotype and the environment:

$$Fitness= P\left( Y=1 | E=1 \right)P(E=1)+P\left( Y=0 \right| E=0)P(E=0)$$

$$Fitness= (P\left( Y=1 | E=1 \right)+P\left( Y=0 \right| E=0))/2$$

We first calculate $P\left( Y=1 | E=1 \right)$, the probability of finding $Y=1$ given that the initial value of $E$ is 1.

$$P\left( Y=1 | E=1 \right)=\frac{\sum_{\{X_{i}\}} P\left( Y=1 \right| \left\{ X_{i} \right\})P\left( \left\{ X_{i} \right\} \right| E=1)}{\sum_{\{X_{i}\}} (P\left( Y=1 \right| \left\{ X_{i} \right\})P\left( \left\{ X_{i} \right\} \right| E=1)+P\left( Y=1 \right| \left\{ X_{i} \right\})P\left( \left\{ X_{i} \right\} \right| E=0))}$$

Denominator is equal to 1 by symmetry. Each $\left\{ X_{i} \right\}$ contains *k* elements 1. Then:

- Summation over all possible $\left\{ X_{i} \right\}$ can be written as a summation over the number *k* of 1 in $\left\{ X_{i} \right\}$.
- then $P\left( \left\{ X_{i} \right\} \right| E=1)=\eta^{k}{(1-\eta)}^{m-k}$ and $P\left( \left\{ X_{i} \right\} \right| E=0)=\eta^{m-k}{(1-\eta)}^{k}$
- and $P\left( Y=1 \right| \{X_{i}\})=Logistic(\beta_{0}+\sum_{i=0}^{m} \beta_{i}X_{i})=Logistic(\beta_{0}+k\tilde{\beta})$
- following a similar reasoning as above, $Logistic\left( \beta_{0}+k\tilde{\beta} \right)=\frac{\eta^{k}{(1-\eta)}^{m-k}}{\eta^{k}{(1-\eta)}^{m-k}+\eta^{m-k}{(1-\eta)}^{k}}$

Therefore,

$$P\left( Y=1 | E=1 \right)=\sum_{k=0}^{m} \binom{m}{k}\frac{\eta^{2k}{(1-\eta)}^{2m-2k}}{\eta^{k}{(1-\eta)}^{m-k}+\eta^{m-k}{(1-\eta)}^{k}}$$

Similarly,

$$P\left( Y=0 | E=0 \right)=1-P\left( Y=1 \right| E=0)$$

$$=1-\sum_{k=0}^{m} \binom{m}{k}\frac{\eta^{m}{(1-\eta)}^{m}}{\eta^{k}{(1-\eta)}^{m-k}+\eta^{m-k}{(1-\eta)}^{k}}$$

Using $\sum_{k=0}^{m} \binom{m}{k}\eta^{k}{(1-\eta)}^{m-k}=1$, we obtain

$$P\left( Y=0 | E=0 \right)=\sum_{k=0}^{m} \binom{m}{k}\frac{\eta^{2k}{(1-\eta)}^{2m-2k}}{\eta^{k}{(1-\eta)}^{m-k}+\eta^{m-k}{(1-\eta)}^{k}}$$

Finally,

$$Fitness=\sum_{k=0}^{m} \binom{m}{k}\frac{\eta^{2k}{(1-\eta)}^{2m-2k}}{\eta^{k}{(1-\eta)}^{m-k}+\eta^{m-k}{(1-\eta)}^{k}}$$

which gives the theoretical fitness for a network with $m$ sensors selected for a history with signal fidelity $\eta$.

The accuracy calculated here is based on the probabilistic expression of the arithmetic mean of the fitness. To calculate the theoretical fitness across the past environments, we need to use the geometric mean. Then the expression becomes:

$$Fitness=exp(\sum_{k=0}^{m} \binom{m}{k}\log\left( \frac{\eta^{k}\left( 1-\eta\right)^{m-k}}{\eta^{k}\left( 1-\eta\right)^{m-k}+\eta^{m-k}\left( 1-\eta\right)^{k}} \right)\eta^{k}\left( 1-\eta\right)^{m-k}+\binom{m}{k}\log\left( 1-\frac{\eta^{k}\left( 1-\eta\right)^{m-k}}{\eta^{k}\left( 1-\eta\right)^{m-k}+\eta^{m-k}\left( 1-\eta\right)^{k}} \right)\eta^{k}\left( 1-\eta\right)^{m-k}$$

## Mutants in Wsp module identified from swarming selection

**Table S1.** Mutations identified in the Wsp system from *fleN*ΔwspF* (see **Fig. 6G**)

| **Gene** | **Mutation*** | **Occurrence** |
| --- | --- | --- |
| wspA | Δ857-859 | 4 |
| wspA | Δ1056-1056 | 1 |
| wspA | Δ1174-1174 | 1 |
| wspA | C->T (A397V) | 1 |
| wspA | C->A (A418E) | 1 |
| wspA | Δ1321-1372 | 1 |
| wspA | ins'A' | 1 |
| wspA | G -> C (V537L) | 1 |
| wspB | T-> G (V53G) | 2 |
| wspB | C->T (Q108*) | 1 |
| wspB | ins'CTCGGCCCGGTCTATGAAGGACCACGGGGACTGGTGCAATGGGTGCGG' | 1 |
| wspB | T-> G (V147G) | 4 |
| wspB | ins'G' | 1 |
| wspB | Δ504-504 | 1 |
| wspC | A -> G (N2D) | 1 |
| wspC | T -> G (L38R) | 1 |
| wspC | G -> T (E54D) | 1 |
| wspC | G -> A (V62M) | 3 |
| wspC | C -> T (R71C) | 1 |
| wspC | T -> C (L82P) | 1 |
| wspC | Δ336-337 | 1 |
| wspC | Δ430-430 | 1 |
| wspC | G -> T (E155*) | 1 |
| wspC | Δ941-951 | 1 |
| wspC | ins'GGCGCTGGCGCACCTGGC' | 1 |
| wspD | T -> G (V123G) | 2 |
| wspD | Δ654-654 | 1 |
| wspE | Δ332-1801 | 1 |
| wspE | Δ477-495 | 3 |
| wspE | T -> G (V194G) | 1 |
| wspE | Δ646-698 | 1 |
| wspE | C -> T (Q325*) | 1 |
| wspE | Δ1353-1400 | 2 |
| wspE | Δ1907-1917 | 2 |
| wspR | Δ162-167 | 3 |
| wspR | ins'CGGCAACC' | 1 |
| wspR | C -> T (P105L) | 1 |
| wspR | A -> G (K108E) | 1 |
| wspR | C -> T (R132W) | 1 |
| wspR | A -> T (E227V) | 1 |
| wspR | G -> C (V258L) | 2 |
| wspR | Δ809-1059 | 1 |
| wspR | Δ885-896 | 4 |

*Δ, deletion with the start and stop position of nucleotides deleted in the gene. ins, insertion with inserted sequence indicated in single quote. SNVs with nucleotide before and after swarming occurs was displayed, followed by change of amino acid residues with position indicated.

## Expanded Material and Methods section

### Phenotypic assays

*P. aeruginosa* starter cultures were inoculated from glycerol stocks and incubated at 37°C with vigorous shaking. In the swarming assay, the cultures were washed twice in sterile phosphate buffer solution (PBS) and then suspended in PBS. 2 μl of washed culture are then inoculated on swarming agar and incubated 37°C for 24h (15). Biofilm was quantified using the crystal violet (CV) method. The overnight culture was diluted 1:60 in rich liquid medium (LB) and continued to shake at 37°C for 3h to reach exponential growth. The culture was then diluted in tryptone broth (TB) liquid medium to OD_600_ of 0.0025, aliquoted to 8 wells of 96-well plate, and incubated at room temperature. After 24h, cells in the supernatant were measured by absorbance at 600 nm (A_600_). The final biofilm was stained using CV which was then dissolved in 33% (v/v) acetic acid and quantified by A_595_. A negative control of only TB served as blank. Biofilm index was calculated [(A_595-_A_595blank_)/(A_600-_A_600blank_)] and normalized to the laboratory strain PA14.

### Biofilm selection in drip-flow reactor

We conducted two runs of the drip-flow bioreactor experiment. In the first run, the flow rate was set as 4ml/10min and the experiment ran for five days. All four parallel channels of the drip-flow reactor were inoculated with hyperswarmer FleN(V178G) (15). In the second run the flow rate was set as 2ml/10min and the experiment ran for seven days. Two channels were inoculated with FleN(V178G) and two with its ancestral isogenic strain PA14. After the runs, the biofilms were collected and sonicated to obtain single cells, which were serially diluted and plated for clone isolation. Colonies were picked with pipette tips and their swarming phenotype was assessed. Three colonies were selected for each group and screened using phenotypic assays.

### Construction of mutants by allelic exchange

The protocol of construction SNVs/indel/inframe deletion of *dipA* and *wspF* mutants was performed as described previously (15). For SNP/indel construction, a ~500 bp target sequence of *dipA* or *wspF* was amplified from either ancestral or evolved genome. A suicide plasmid introduced the amplified allele into targeted *P. aeruginosa* genome through homologous recombination (16). One copy of the sequence was excised out by sucrose counter selection and the successful clones were confirmed by Sanger sequencing (Genewiz Inc.) and swarming morphology. For in-frame deletion of *wspF*, an extra splicing overlap extension (SOE) step was performed prior to ligation to plasmid, which left a 51 bp fragment with the start and end of *wspF* gene. The fragment was then used for allelic exchange to substitute the intact *wspF* sequence in *P. aeruginosa* genome.

**Table S2.** Primer sequences used in this study.

| **Primer name** | **Primer sequence^a, b^** | **Usage** |
| --- | --- | --- |
| DFR1-4-14_1up | CTA CGA AGA CAT CAC GCA GAA C | Sequencing primer to confirm *dipA** SNP |
| DFR1-4-14_1down | CAA TGC TTT CGC CCT TTT CCA C |  |
|  |  |  |
| DFR1-4-14sw_fd | GGG GAC AAG TTT GTA CAA AAA AGC AGG CTA CTG GTG GAA TAC GTC AAT CCG AG | making allelic replacement of *dipA** mutation |
| DFR1-4-14sw_rv | GGG GAC CAC TTT GTA CAA GAA AGC TGG GTA GGA TCA TGC CTT TTT CCG GAT G |  |
|  |  |  |
| 66320_seq3_up | GCA GAC CCT GAT GAA GTA CGC | sequencing primer to confirm *dipA*** SNP |
| 66320_seq3_dw2 | TCG CTT TTC TTG GTG AGG ATT TC |  |
|  |  |  |
| 66320_sw2_fw | GGG GAC AAG TTT GTA CAA AAA AGC AGG CTA CCA TCC GGA AAA AGG CAT GAT | For making allelic replacement of *dipA*** mutation |
| 66320_sw2_rv | GGG GAC CAC TTT GTA CAA GAA AGC TGG GTA CGG ATG AGC ACA GAG AGT ACA G |  |
|  |  |  |
| PA16480 _seq1_up | CAG CAT GTC GAC GAG GTA TTC | sequencing primer to confirm *wspF** indel |
| PA16480 _seq1_dn | CCT CGT ATC AAA TTG AAT GCT G |  |
| 16480sw_fw | GGG GAC AAG TTT GTA CAA AAA AGC AGG CTA CCA GCA TGT CGA CGA GGT ATT C | For making allelic replacement of *wspF** mutation |
| 16480sw_rv | GGG GAC CAC TTT GTA CAA GAA AGC TGG GTA CCT CGT ATC AAA TTG AAT GCT G |  |
|  |  |  |
| wspF_out_fw | GTC GAG GAC CTC CTG CAT TC | sequencing primer to confirm Δ*wspF* deletion |
| wspF_out_rv | CTT GGT CGA CAG GAC GAT GAT |  |
| wspF_Upfw1 | GGG GAC AAG TTT GTA CAA AAA AGC AGG CTA C GAGCTGGAACGCAAGCTCCT | Primer pair to amplify the starting region of *wspF* gene |
| wspF_Uprv1 | CGG CTA ATC GAA TAC CTC CGC CAG CGG CATGTCATTGACGATTCCGATC | Also for SOEing of starting and ending region of *wspF* gene |
| wspF_Dnfw1 | CTGGCGGAGGTATTCGATTAGCCG | Primer pair to amplify the ending region of *wspF* gene |
| wspF_Dnrv1 | GGG GAC CAC TTT GTA CAA GAA AGC TGG GTA GATCACCGTCGGCTTGATCTGG |  |
|  |  |  |
| wspA_seq_fw1 | GAG GAA CTC AAG GGC CTG GT | sequencing primer to comfirm *wspA* mutation |
| wspA_seq_rv1 | GAG AGC AGG TTG GTC TGG TC |  |
|  |  |  |
| wspR_seq_fw1 | CAG CAT TCA ATT TGA TAC GAG GGG | sequencing primer to comfirm *wspR* mutation |
| wspR_seq_rv1 | ATG TAC GAA CGC GAG TGG TAG |  |
|  |  |  |
| wsp_fw2 | TCT TCT CAG AAA AGT CTG GCT TGC | to amplify *wsp* locus to identify mutation in swarming evolved *wsp* mutants |
| wsp_rv2 | GCG ATC ATG CTT CTG ATC GTA GTC |  |

a. Gateway sequence for upstream (forward) primer is marked with underline

b. Gateway sequence for downstream (forward) primer is marked with underline

### Biofilm colony morphology and c-di-GMP quantification

Colony growth for Congo red quantification of extracellular polymers and bulk c-di-GMP levels were performed as previously described (17, 18) with slight modifications: To obtain a colony biofilm, overnight culture of *P. aeruginosa* cells were washed and diluted to OD_600_ = 1. Four spots of 10 μL cell were spotted on 1% agar / 1% tryptone plates with (in the Congo red binding assay) or without (in the c-di-GMP assay) dye (20 mg/L Coomassie blue, 40 mg/L Congo red) and incubated at room temperature for 3 days to form a dense colony. For c-di-GMP quantification, colonies were harvested and washed in TB and extracted with methanol/acetonitrile/water (40:40:20) with 0.1 N formic acid at -20°C for 1h. The cell extract was then pelleted by centrifugation at 13,000 rpm for 5 min at 4°C. Two hundred microliters of supernatant were transferred and neutralized with 8 μl of 15% NH_4_HCO_3_, dried with a speed vacuum and re-suspended in 200 μl phaseA (10mM tributylamine +15mM Acetic acid). Samples were quantified in Mass Spectrometry Facility of Michigan State University with electrospray ionization (ESI) analysis with Quattro Premier XE LC/MS/MS. The final concentration of c-di-GMP was normalized using cell dry weight.

### PacBio sequencing and *de novo* assembly of clinical *P. aeruginosa* strains

Genomic DNA of clinical *P. aeruginosa* strains was extracted using the Genomic DNA Buffer Set and Genomic tip (Qiagen). For each genomic sample, 3.4 to 6.1 µg of high-quality genomic DNA was used for library preparation and sheared to ~20 kb. The final SMRTbell library was selected in the range of 7,000 bp~50,000 bp with 0.45X AMPure magnetic bead exchange and Sage Science Blue Pippin 0.75% agarose cassettes. The sequencing was done on the RSII machine at a concentration of 100-150 pM and configured for a 180-minute continuous sequencing protocol. For assembly, the reads were processed using a *de novo* assembly protocol with the Hierarchical Genome Assembly Process (HGAP) v2.1 SMRTportal assembly pipeline filtering at 0.80 RQ, 500 bp sub-readlength, and standard pre-assembly pipeline parameters.   The HGAP assembler uses the DAGCon-based pre-assembler module and Quiver for post-assembly consensus validation. The median reading depth of the clinical strains is 79x with a range of 30x ~179x.

### Computational analysis of genomic sequences

The assembled PacBio genomic sequences were analyzed with a custom bioinformatics pipeline and with the PATRIC system (19). Open reading frames where identified de novo for every genome and orthologs where identified using reciprocal blast. The ortholog sequences were store in a postgresql database called SpringDB which was used for efficient identification of core genomes and genomic variants. Downstream analyses were carried out in Matlab version 2014b, the Mathworks^TM^ with the *Bioinformatics* and *Statistics and Machine Learning* toolboxes.

### High time-resolution growth and GFP expression monitoring

The experimental protocol and data analysis follow Boyle et al (20). Bacterial strains were inoculated into LB medium and cultured overnight with shaking at 37°C. Cells were harvested and washed with sterile phosphate buffer solution (PBS) twice and diluted into OD_600_ = 0.0025 in fresh casamino acid medium (5g/L casamino acid, 1 mM MgSO_4_, 0.1 mM CaCl_2_ and 1X buffer (12 g/L Na_2_HPO_4_ (Fisher Scientific), 15 g/L KH_2_PO_4_ (Fisher Scientific) and 2.5 g/L NaCl, pH6.7)). OD600 and GFP signals (W_excitation_ = 488nm, W_emission_ = 525nm) were measured every 10 minutes using a Tecan M1000 plate reader (Mannedor, Switzerland). Growth curves were aligned to make sure that they are compared at the same growth phase. The PfliC-GFP signal was compared through the strains when they transited from exponential phase to early stationary phase (20).

## Supplementary references

1. Martins EP & Hansen TF (1997) Phylogenies and the comparative method: a general approach to incorporating phylogenetic information into the analysis of interspecific data. *American Naturalist*:646-667.

2. Pease C & Bull J (1988) A critique of methods for measuring life history trade‐offs. *Journal of Evolutionary Biology* 1(4):293-303.

3. Lande R (1979) Quantitative genetic analysis of multivariate evolution, applied to brain: body size allometry. *Evolution*:402-416.

4. Eyre-Walker A (2010) Genetic architecture of a complex trait and its implications for fitness and genome-wide association studies. *Proceedings of the National Academy of Sciences* 107(suppl 1):1752-1756.

5. Maddison WP & FitzJohn RG (2015) The unsolved challenge to phylogenetic correlation tests for categorical characters. *Syst Biol* 64(1):127-136.

6. Felsenstein J (1985) Phylogenies and the comparative method. *American Naturalist*:1-15.

7. Tibshirani R (1996) Regression Shrinkage and Selection via the Lasso. *Journal of the Royal Statistical Society. Series B (Methodological)* 58(1):267-288.

8. Perkins TJ & Swain PS (2009) Strategies for cellular decision-making. *Molecular systems biology* 5:326.

9. Matsuyama BY*, et al.* (2016) Mechanistic insights into c-di-GMP–dependent control of the biofilm regulator FleQ from *Pseudomonas aeruginosa*. *Proceedings of the National Academy of Sciences* 113(2):E209-E218.

10. Baraquet C & Harwood CS (2013) Cyclic diguanosine monophosphate represses bacterial flagella synthesis by interacting with the Walker A motif of the enhancer-binding protein FleQ. *Proceedings of the National Academy of Sciences* 110(46):18478-18483.

11. Friedlander T, Mayo AE, Tlusty T, & Alon U (2015) Evolution of Bow-Tie Architectures in Biology. *PLoS Comput Biol* 11(3):e1004055.

12. Kussell E & Leibler S (2005) Phenotypic diversity, population growth, and information in fluctuating environments. *Science* 309(5743):2075-2078.

13. Alpaydin E (2014) Introduction to machine learning. (MIT press).

14. Allen RC, Popat R, Diggle SP, & Brown SP (2014) Targeting virulence: can we make evolution-proof drugs? *Nat Rev Micro* 12(4):300-308.

15. van Ditmarsch D*, et al.* (2013) Convergent evolution of hyperswarming leads to impaired biofilm formation in pathogenic bacteria. *Cell reports* 4(4):697-708.

16. Shanks RM, Caiazza NC, Hinsa SM, Toutain CM, & O'Toole GA (2006) *Saccharomyces cerevisiae*-based molecular tool kit for manipulation of genes from gram-negative bacteria. *Appl Environ Microbiol* 72(7):5027-5036.

17. Kempes CP, Okegbe C, Mears-Clarke Z, Follows MJ, & Dietrich LE (2014) Morphological optimization for access to dual oxidants in biofilms. *Proc Natl Acad Sci U S A* 111(1):208-213.

18. Monds RD*, et al.* (2010) Di-adenosine tetraphosphate (Ap4A) metabolism impacts biofilm formation by *Pseudomonas fluorescens* via modulation of c-di-GMP-dependent pathways. *Journal of bacteriology* 192(12):3011-3023.

19. Wattam AR*, et al.* (2014) PATRIC, the bacterial bioinformatics database and analysis resource. *Nucleic acids research* 42(Database issue):D581-591.

20. Boyle KE, Monaco H, van Ditmarsch D, Deforet M, & Xavier JB (2015) Integration of Metabolic and Quorum Sensing Signals Governing the Decision to Cooperate in a Bacterial Social Trait. *PLoS Comput Biol* 11(6):e1004279.
